# Metal catalyzed C–H functionalization on triazole rings

**DOI:** 10.1039/d2ra05697f

**Published:** 2022-09-28

**Authors:** Anushka Koranne, Khushboo Kurrey, Prashant Kumar, Sangeeta Gupta, Vikesh Kumar Jha, Rangnath Ravi, Prasanta Kumar Sahu, Anamika, Abadh Kishor Jha

**Affiliations:** Govt. Shivnath Science College Gaurav Path Rajnandgaon 491441 Chhattisgarh India awadhbrave@gmail.com; RRM PG College Surajpur Chhattisgarh India; Shivaji College, University of Delhi Delhi India; Jawaharlal Nehru University New Delhi India

## Abstract

The present review covers advancement in the area of C–H functionalization on triazole rings, by utilizing various substrates with palladium or copper as catalysts, and resulting in the development of various substituted 1,2,3- and 1,2,4-triazoles. Synthesis of these substituted compounds is necessary from the perspective of pharmaceutical, medicinal, and materials chemistry.

## Abstract 2

The present review covers advancement in the area of C–H functionalization on triazole rings, by utilizing various substrates with palladium or copper as catalysts, and resulting in the development of various substituted 1,2,3- and 1,2,4-triazoles.

## Graphical abstract

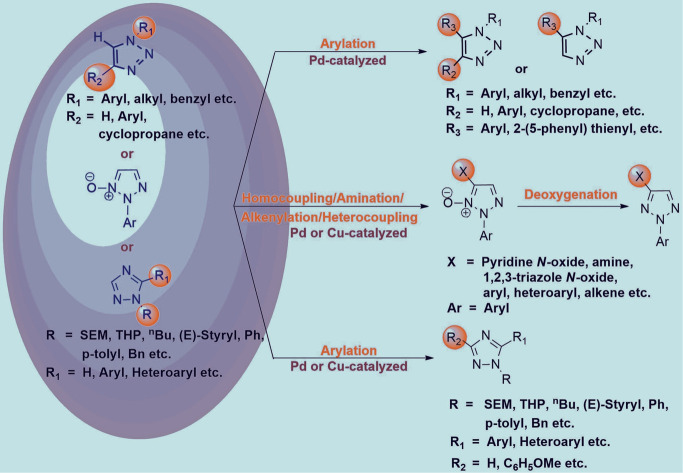

## 1. Introduction

Since the discovery of the Ullmann coupling reaction in the early twentieth century, there has been an intense and sustained research on the synthesis of biaryl compounds, which appears to be an intriguing branch of organic chemistry having a broad range of applications. Among them, 5-membered heteroaryls, especially triazoles and their derivatives have surfaced as a significant heterocyclic class with their rapidly increasing applications in medicinal,^1^ materials^2^ as well as other chemistry,^1^ including their importance in anticancer drugs and antipsychotics.^2^

Over the past years, the cross-dehydrogenative coupling and C–H bond activation, owing to its synthetic efficiency and atom economy, has attracted a tremendous amount of attention in modern organic synthesis.^3^ Triazoles and their derivatives synthesised by various methods have become the primary structural motifs of several compounds having their applicability in a wide range of areas including and not limited to medicinal chemistry, agriculture, and materials science.^1,2,4^ In 2005, Fagnou's group and other groups introduced triazoles and several other heterocyclic *N*-oxides as stable and readily available substrates for undergoing cross-coupling or heterocoupling reactions.^5^ Since then, in the last two decades, metal-catalyzed C–H bond activation on triazoles has evolved as an effective tool for the formation of desired products as these reactions involve C–H activation on the triazole ring, shorten the synthetic routes, utilise easily available and low cost starting materials and are a better choice from an economic and environmental perspective. The synthesis of these products *i.e.* diverse 1,2,3-triazole derivatives has become a very crucial area of research due to their myriad of applications in pharmaceutical,^6^ medicinal^1^ and materials chemistry.^2^ The present review summarises palladium and copper catalyzed C–H functionalization on triazole rings to produce substituted triazoles & covers advancement in this field of research from June 2010 to June 2022.

## 2. Palladium-catalyzed C–H functionalization on triazole rings

### 2.1. Palladium-catalyzed C–H functionalization on 1,2,4-triazole rings

**Scheme 1:**
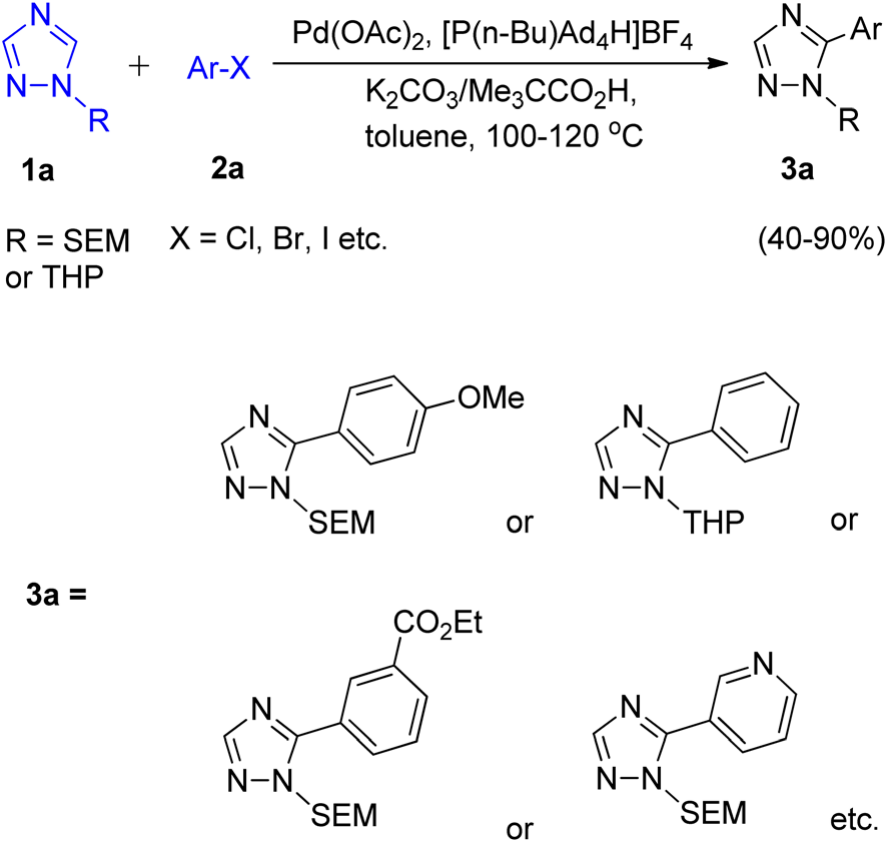
Palladium catalyzed arylation of 1-substituted-1,2,4-triazoles.

In order to overcome the limitation of C–H arylation of 1,2,4-triazole to only 1-methyltriazoles, a novel strategy for the formation of complex arylated 1,2,4-triazole was developed by Dalibor Sames and co-workers in 2012.^7^ A benchtop procedure for the direct C–H arylation of 1-substituted 1,2,4-triazole was firstly developed by employing air-stable phosphonium salts (Scheme 1).

**Scheme 2:**
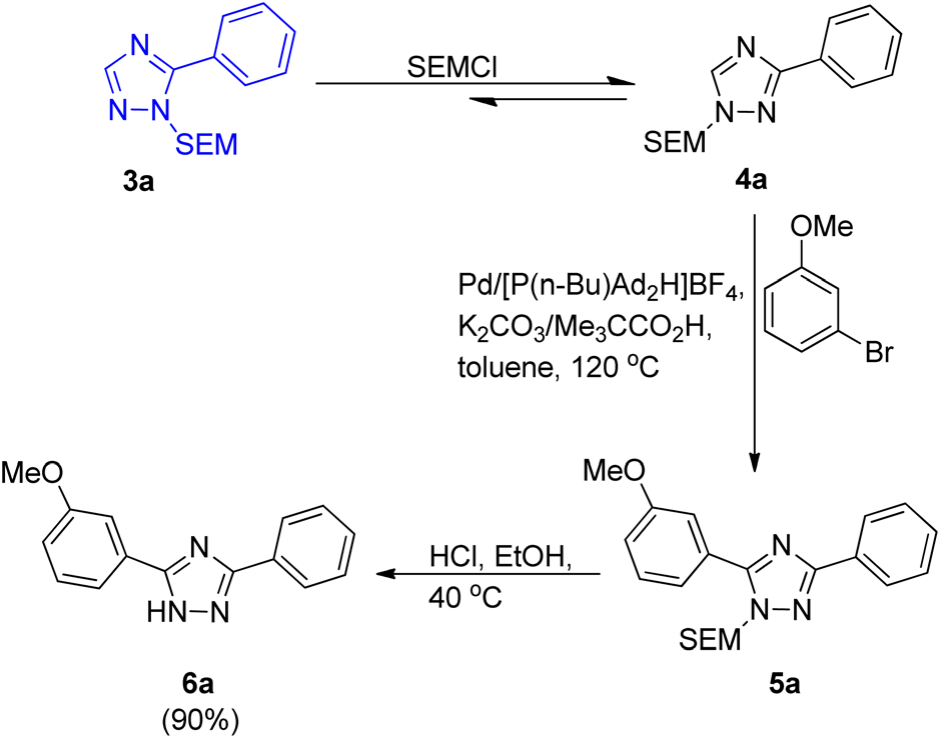
SEM-switch and arylation.

Although the utilization of more soluble and bulkier carboxylic acid as co-catalyst showed higher efficiency as compared to pivalic acid, giving modest benefit, pivalic acid being easily available and inexpensive was employed for analysis of reaction parameters. After analyzing various reaction parameters, Pd(OAc)_2_ (5 mol%) as catalyst, [P(*n*-Bu)Ad_2_H]BF_4_ (10 mol%) as ligand, carboxylic acid (0.3 equiv.) as co-catalyst, toluene (1.0 M) as solvent and potassium carbonate (3.0 equiv.) in stoichiometric amount as base was found to be very essential in order to attain maximum efficiency at 120 °C. This palladium-catalyzed coupling reaction gave the arylated product in moderate to good yield (40–90%) for SEM (trimethylsilyl ethoxy methyl) as well as for THP (tetrahydropyranyl) group. Apart from iodobenzene which required slight changes in the catalytic system (utilizing Pd(PPh_3_)_2_Cl_2_ as ligand and Ag_2_CO_3_ as base), both bromobenzene and chlorobenzene coupled with 1-substituted triazole under the optimized conditions. A number of functional groups such as dimethylamino, pyridyl, ester, and methoxy groups, were tolerated by bromoarenes used for arylation. It was found that analogous to the SEM switch of diazoles,^7^ the SEM group attached to these triazoles can easily undergo a SEM-switch from N-1 to N-2 in presence of SEM-Cl (catalytic amount) to form the least sterically hindered 3-aryltriazole 4a. Thus, this process provides a simple route to convert the unreactive C-3 position to a reactive C-5 position which then on subsequent arylation (reaction conditions similar to as shown in Scheme 1) resulted in the generation of diaryltriazole 5a (Scheme 2). The same switch is also applicable for the THP group. The SEM and THP groups of the triazole can be easily separated by applying mild acidic conditions.

**Scheme 3:**
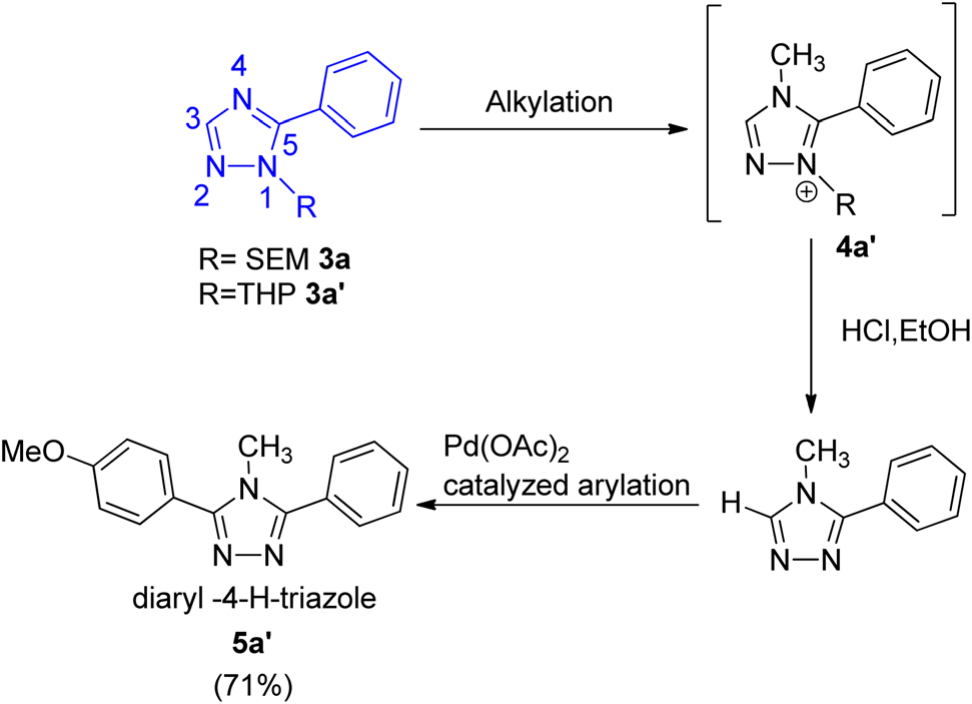
*Trans-N*-alkylation and arylation of 1-SEM- and 1-THP-5-aryltriazole.

The protocol thus developed also gave 4-methyl 3,5-diaryltriazole (Scheme 3; 5a′) in good yield (71%) when the *trans-N*-arylation and alkylation of 1-SEM- and 1-THP-5-aryltriazole (Scheme 3; 3a and 3a′) was carried out, representing the 1st catalytic C–H arylation of 4-alkyltriazoles (Scheme 3; 4a′). Thus, the protocol was applicable for arylation of 1-alkyltriazoles as well as for the arylation of regioisomeric 4-alkyltriazoles.

### 2.2. Palladium-catalyzed C–H functionalization on 1,2,3-triazole rings

Chunxiang Kuang *et al.*, in 2013, reported a highly chemoselective, stereoselective and regioselective palladium-catalyzed synthesis of 2-substituted 4-alkenyl- and 4-aryl-1,2,3-triazoles *via* C–H functionalization of 1,2,3-triazole *N*-oxides.^8^ The activity of 2-substituted 1,2,3-triazole *N*-oxides at C-5 directs the alkenylation and arylation to occur preferably at C-5. These two strategies, one being site selective alkenylation and the other being direct cross-coupling of 2-substituted 1,2,3-triazole *N*-oxides with inactivated arenes, were the two new protocols given by them.

**Scheme 4:**
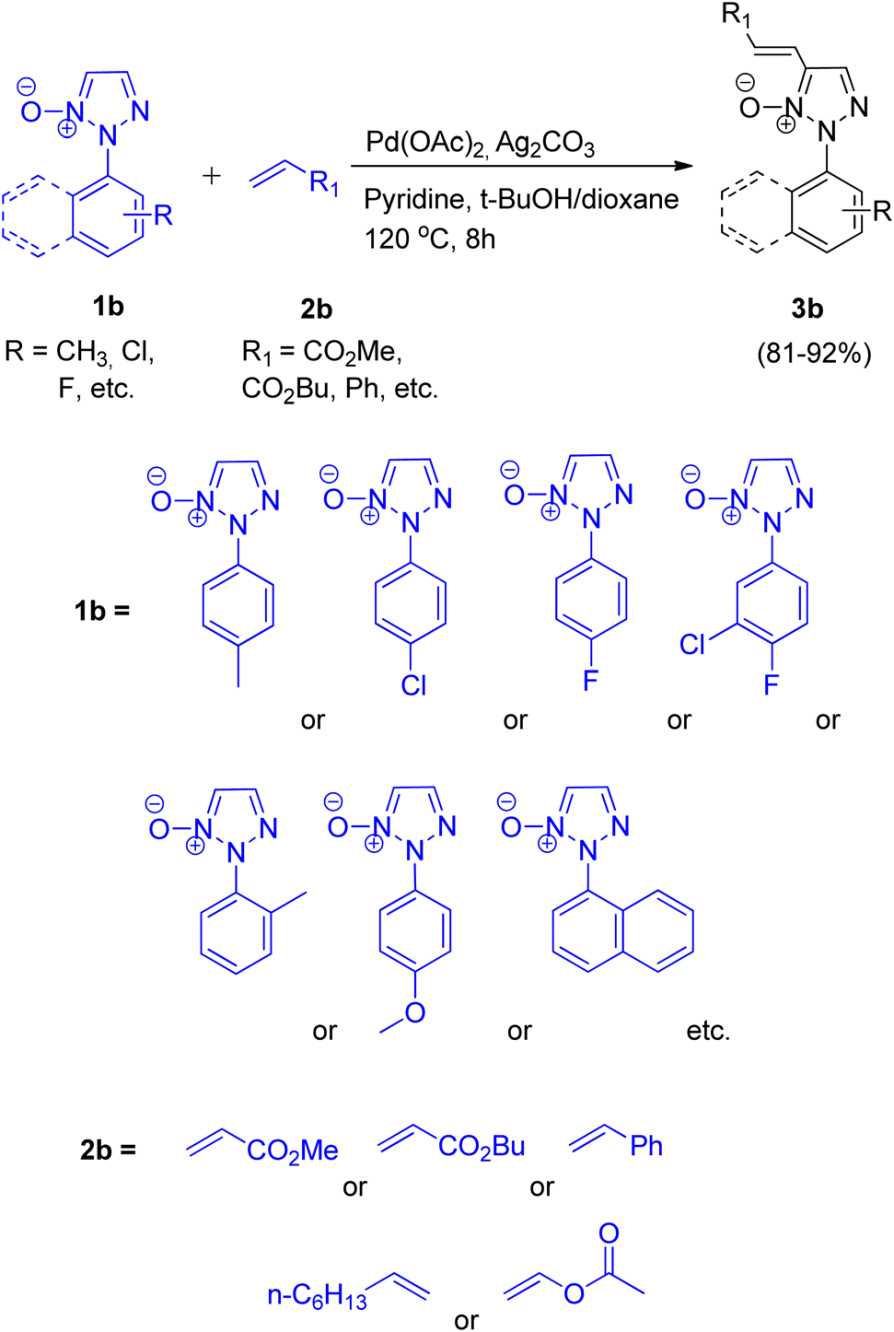
Alkenylation of 2-substituted 1,2,3-triazole *N*-oxides.

The optimum reaction condition for the reaction between 2-substituted 1,2,3-triazole *N*-oxide (1b) and alkene (2b) involved employing Pd(OAc)_2_ (5 mol%) in combination with pyridine (2.0 equiv.), Ag_2_CO_3_ (1.5 equiv.) and *t*-BuOH with dioxane as solvent (Scheme 4). The electronic environment of the substituent present on *N*-oxide (1b) did not affect the overall yield (81–92%) of the desired product 3b. Under similar reaction conditions, the reaction of 2-substituted 1,2,3-triazole *N*-oxide (1b) with vinyl acetate and 1-octene resulted in formation of isomeric alkenylated products in high yields.

**Scheme 5:**
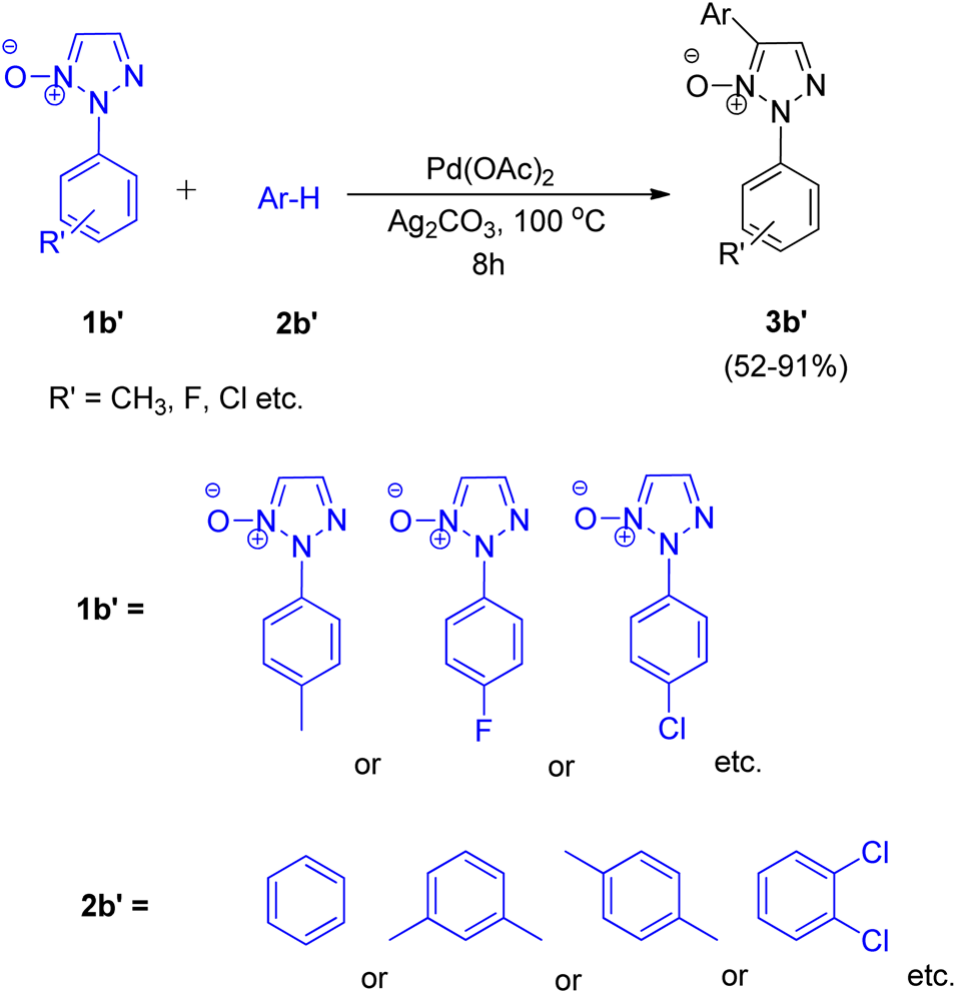
Coupling of 2-substituted 1,2,3-triazole *N*-oxide (1b′) with arenes.

To their surprise, upon carrying out the alkenylation reaction in benzene, in addition to the desired alkenylated compound (3b), 2-substituted 5-aryl-1,2,3-triazole *N*-oxide (3b′) was obtained as a side product. Encouraged by this result, they explored the arylation reaction of 2-substituted 1,2,3-triazole *N*-oxide (Scheme 5) at C5 position which proceeded with good yields (52–91%) of the desired product (3b′). Various screening experiments performed using 1b′ and benzene revealed that presence of 40 equiv. of benzene, 5 mol% of Pd(OAc)_2_, 1.5 equiv. Ag_2_CO_3_ and 100 °C temperature was the required reaction conditions for the arylation of *N*-oxide to proceed smoothly. This protocol was also applied for the coupling reaction of 2-substituted 1,2,3-triazole *N*-oxides with arenes such as *m*-xylene, *p*-xylene, and 1,2-dichlorobenzene, *etc.* (Scheme 5) and the arylated products were obtained with moderate to good yield (52–91%).

**Scheme 6:**
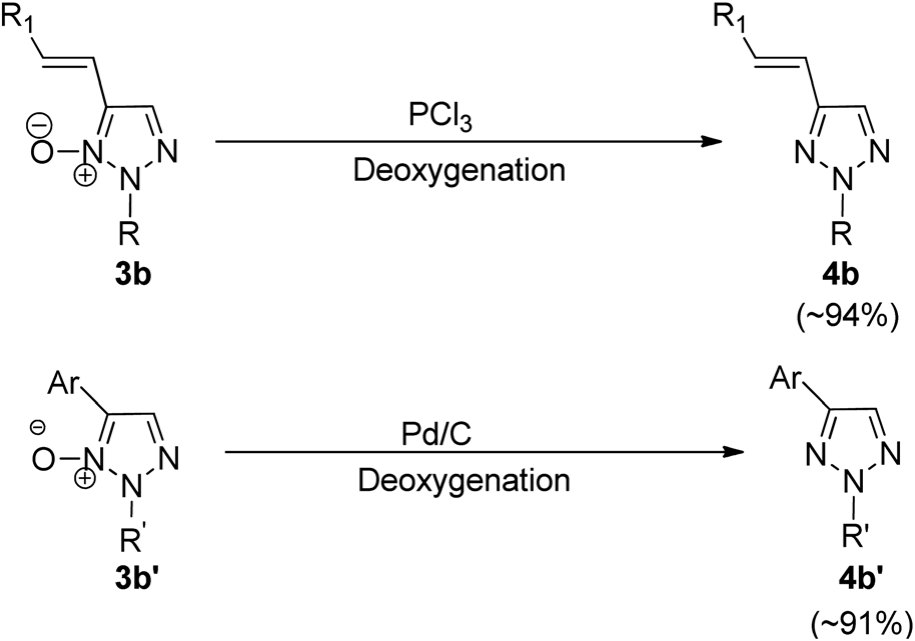
Deoxygenation of 2-substituted 1,2,3-triazole *N*-oxides.

Both alkenylated (3b) and arylated products (3b′) were deoxygenated (Scheme 6) in order to obtain 2-substituted 4-alkenyl (4b) and 4-aryl-1,2,3-triazoles (4b′) respectively. The deoxygenation can be carried out either by using Pd/C and NaBH_4_ in methanol at room temperature for 20 h or by utilizing PCl_3_ in DCM at 60 °C for 1 h to obtain the desired products (3b′ and 4b′) with upto 94% of the isolated yield (Scheme 6).

**Scheme 7:**
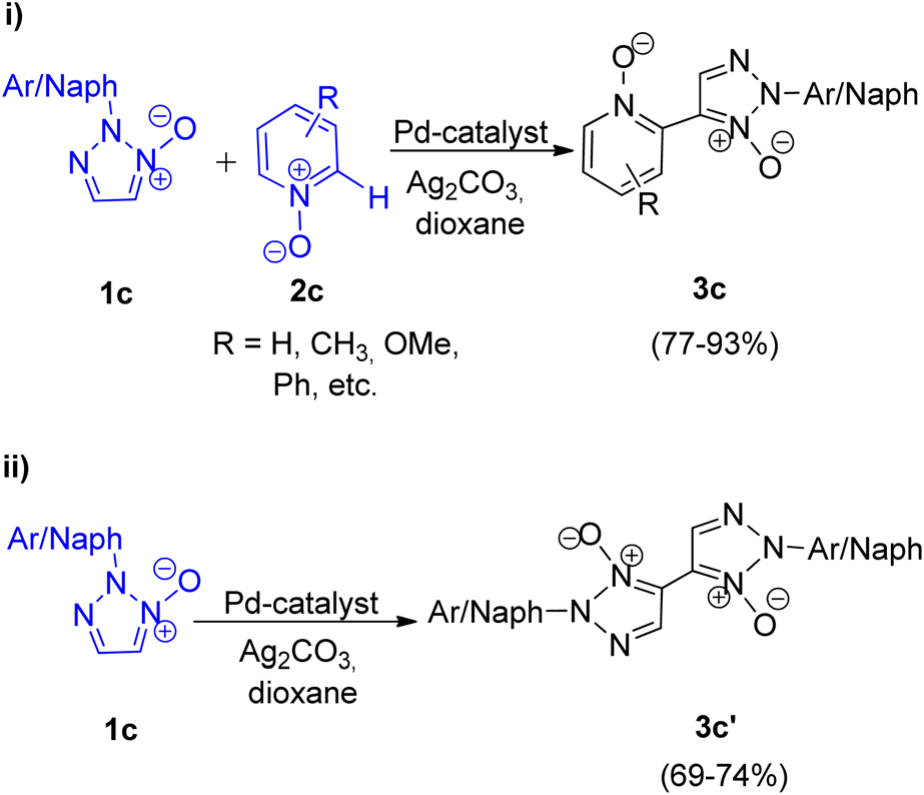
(i) Coupling of 2-aryl-1,2,3-triazole *N*-oxides with other heterocyclic *N*-oxide, (ii) homocoupling of 2-aryl-1,2,3-triazole *N*-oxides.

They also gave the first report on palladium catalyzed homocoupling of 2-substituted 1,2,3-triazole *N*-oxides and oxidative cross-coupling between 2-substituted 1,2,3-triazole *N*-oxides and other heterocyclic *N*-oxides through a 2-fold C–H activation in 2013.^9^ This protocol provided a rare example for the generation of unsymmetrical (Scheme 7; i) and symmetrical (Scheme 7; ii) biheterocyclic *N*,*N*′-dioxides.

**Scheme 8:**
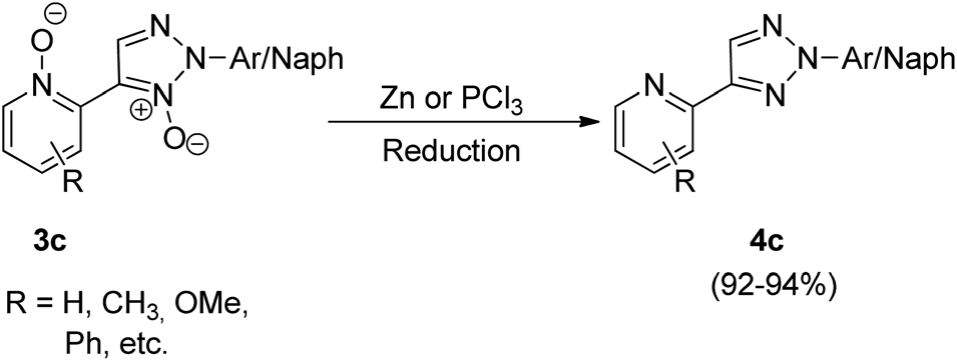
Deoxygenation for biheterocyclic *N*,*N*-dioxides.

The *N*,*N*-dioxides formed in this reaction can be then reduced to obtain the desired biheteroaryl molecule using Zn or PCl_3_ (4c) (Scheme 8). The reaction parameters such as presence of an oxidant, temperature and solvent played an important role in the efficiency of the reaction. Among the various oxidants screened such as Ag_2_CO_3,_ Ag_2_O, AgOAc, Cu(OAc)_2_·3H_2_O, AgNO_3_ and Ag_2_SO_4_, to achieve maximum yield, Ag_2_CO_3_ proved to be the most efficient one. Similarly while the use of solvents such as toluene, DMSO, NMP resulted in good yield of the desired products, the maximum yield (91%) was obtained by using 1,4-dioxane. Thus by using Ag_2_CO_3_ (2 equiv.) as an oxidant in 1,4-dioxane as solvent and Pd(OAc)_2_ (5 mol%) as catalyst at 120 °C, the optimum yield was achieved with complete regioselectivity for oxidative cross-coupling reaction (77–93% yield), however for homocoupling reaction, the yield was very poor which was then increased from 27–30% to 69–74% by adding 0.5 mmol of pyridine as base.

The oxidative cross-coupling reaction takes almost half the time to get completed (24 h) as compared to the homocoupling reaction (48 h). The electronic environment created by the presence of electron-withdrawing and electron-donating groups on the aryl ring of *N*-oxides did not impose much effect on the reaction yield. The approach could also be used with other substituted pyridine *N*-oxides (2c). The desired product can then be obtained in good yield (92–94%) by reduction of the cross-coupling and homocoupling product by utilizing 5 equiv. of Zn with NH_4_Cl, in THF at 70 °C for 2 h or by utilizing 1.2 equiv. of PCl_3_ in CH_2_Cl_2_ at 50 °C for 4 h (Scheme 8).

**Fig. 1:**
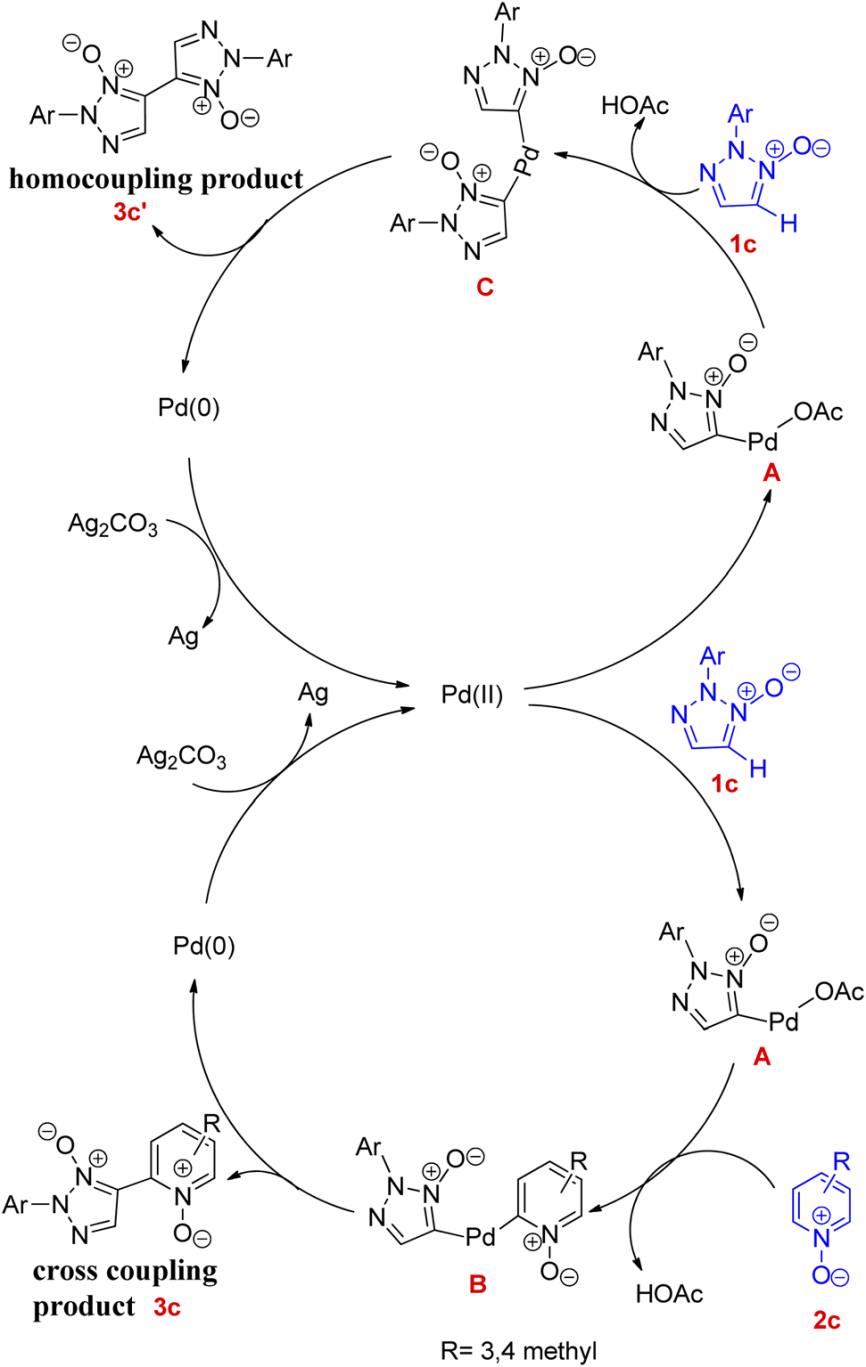
Plausible catalytic cycle for the homocoupling and cross-coupling reaction.

A plausible mechanism for this reaction has been proposed (Fig. 1) which is believed to proceed *via* the formation of intermediate C for homocoupling reaction and intermediate B for cross-coupling reaction.

Both of these coupling reactions start with abstraction of C-5 hydrogen, for the formation of palladium(ii) intermediate (A) *via* reaction of 2-substituted 1,2,3-triazole *N*-oxides with Pd(OAc)_2_. A bisheteroarylpalladium species (B) gets formed by the reaction of this organopalladium species (A) with pyridine *N*-oxide. In a similar way during homocoupling reaction, when this organopalladium species reacts with 1c the intermediate C gets formed. Both of these intermediates *i.e.*B and C undergo reductive elimination reaction to afford the corresponding cross-coupling and homocoupling products 3c and 3c′ respectively.

The introduction of pyridine in homocoupling reaction, considering its usefulness as an activator or a base during the oxidative CH/CH coupling of heteroarenes *N*-oxides,^9^ enhanced the reaction activity and also increased the rate of formation of intermediate C. However, further studies are required to completely understand the mechanism. A similar kind of reaction was developed by them later in 2014, utilising 2,6-lutidine as a ligand (Scheme 11).^11^

**Scheme 9:**
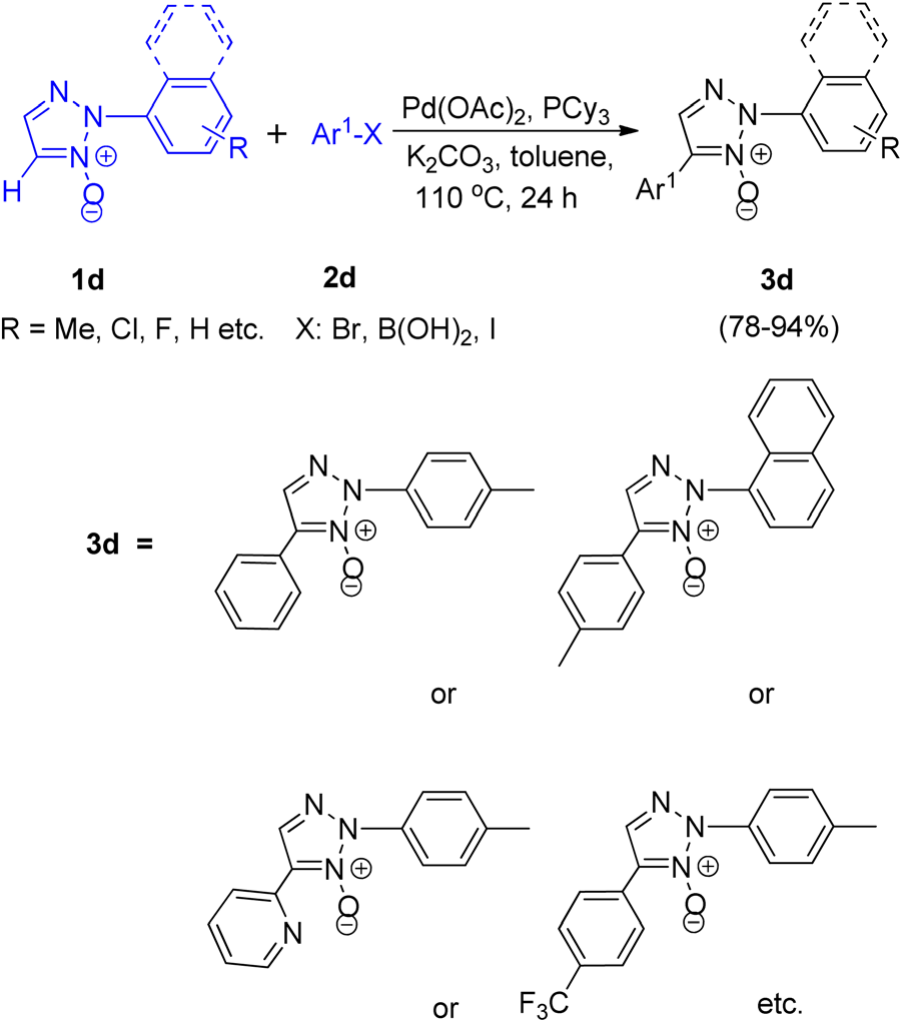
Arylation of 2-aryl-1,2,3-triazoles *N*-oxides at the C-5 position.

**Scheme 10:**
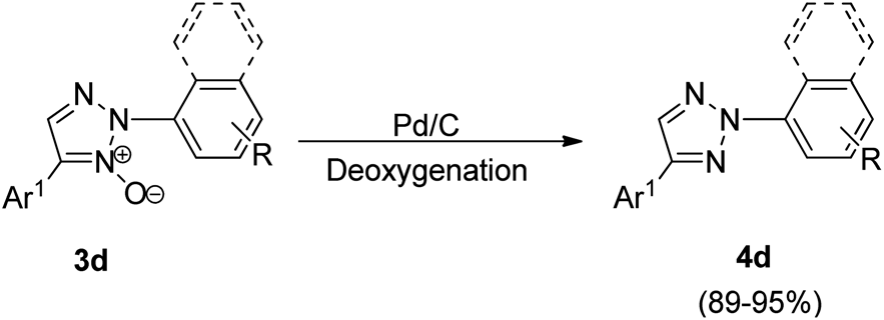
Deoxygenation for 1,2,3-triazole *N*-oxides.

Later on in the same year, formation of 2,4-disubstituted 1,2,3-triazoles by utilising direct coupling between 2-aryl-1,2,3-triazole *N*-oxides and Ar-X [X: Br, B(OH)_2_, I] was also developed by them (Scheme 9).^10^ The desired product was obtained in high yield after the deoxygenation of 3d (Scheme 10).

Various experiments performed to obtain the optimized reaction condition showed that as compared to PtBu_3_–HBF_4_ and PPh_3_, PCy_3_ as ligand was more effective and K_2_CO_3_ as base acted more efficiently than Et_3_N or pyridine. However, apart from the presence of a base, a ligand and the requirement of palladium as a catalyst and toluene as solvent, the existence of high temperature (110 °C) was a prerequisite for the reaction to undergo completion in order to yield the required product (Scheme 9).

**Fig. 2:**
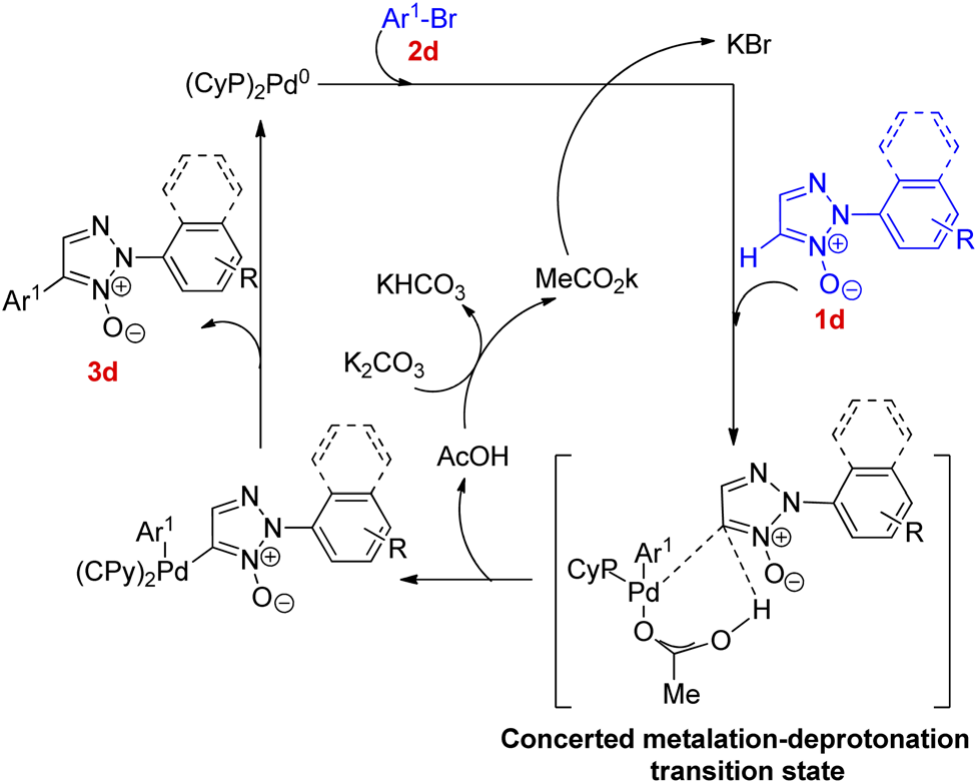
Possible reaction pathway for coupling between 2-aryl-1,2,3-triazole *N*-oxides and Ar^1^-Br involving a concerted metalation-deprotonation step.

Thus the optimized condition for this protocol employed Pd(OAc)_2_ (5.0 mol%) as catalyst, PCy_3_ (5.0 mol%) as ligand, K_2_CO_3_ (1.0 mmol) as base in toluene, at 80 °C for 24 h for synthesizing 2,4-disubstituted 1,2,3-triazoles. The presence of electron rich and electron poor groups on aryl halide (2d) resulted in moderate to good yield (78–94%) of the product. This palladium catalyzed direct arylation reaction has been proposed to proceed *via* a mechanism involving concerted metalation deprotonation (CMD) step for the C–H activation process (Fig. 2). The pathway is expected to hold similarity with the mechanism followed during direct arylation reaction of pyridine *N*-oxide.^10^

The product of interest *i.e.* 2,4-disubstituted 1,2,3-triazole (4d), was then conveniently obtained by deoxygenation of the resulting arylated *N*-oxides (3d) using palladium catalyzed reduction with ammonium formate in methanol at 50 °C for 8 h in as high as 95% yield (Scheme 10).

**Scheme 11:**
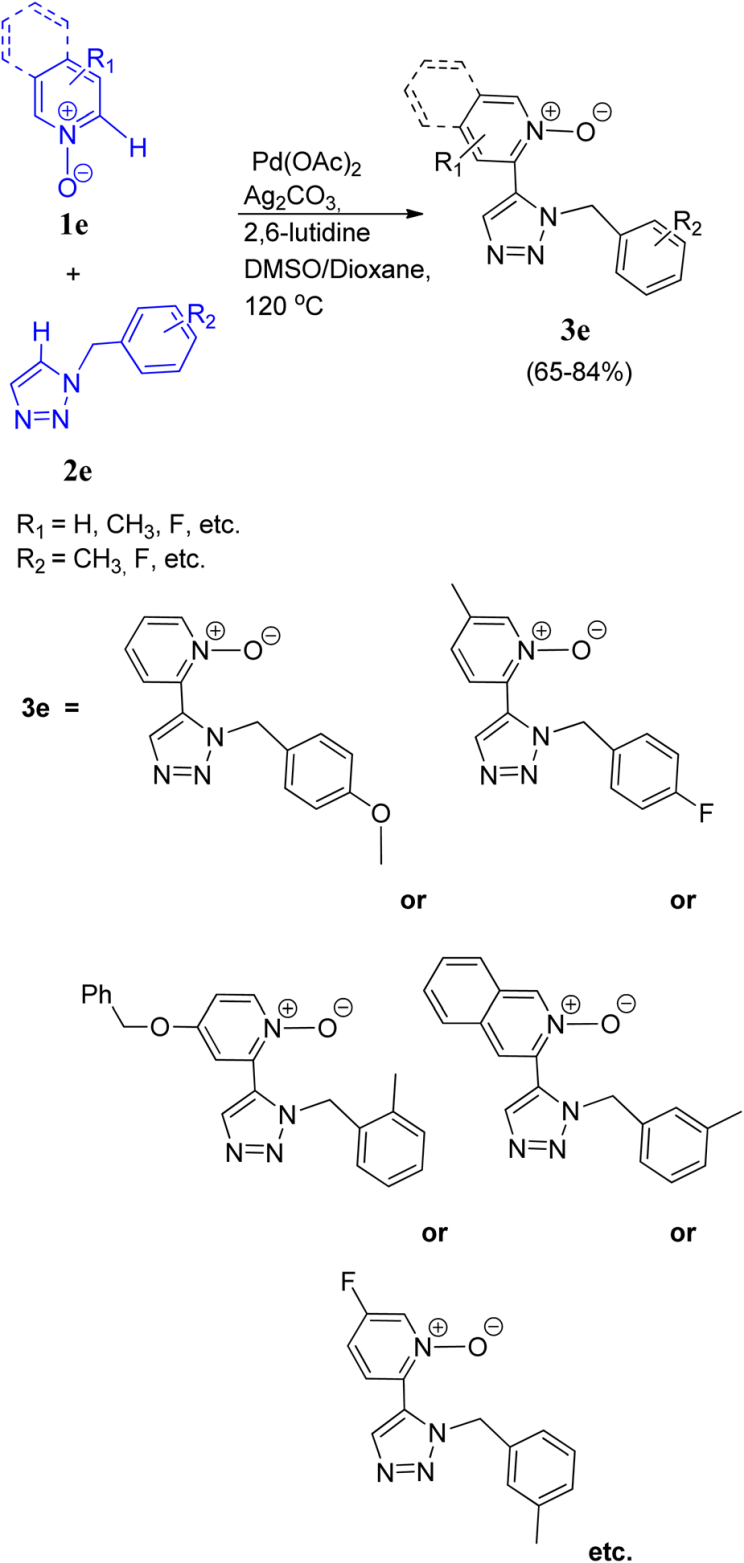
Cross-coupling of pyridine *N*-oxides with 1-substituted-1,2,3-triazole.

Chunxiang Kuang and co-workers^11^ (2014) also developed a site selective CH/CH oxidative cross-coupling between pyridine *N*-oxides (1e) and five membered heterocycles such as 1-benzyl-1,2,3-triazole (2e) and other derivatives *via* a highly efficient two-fold C–H activation (Scheme 11). During optimization it was found that utilization of DMSO with dioxane as solvent resulted in the highest yield of product (65–84%), instead of using them separately even at high temperature (160 °C) or using DMF or NMP. Similarly 2,6-lutidine was found to act superior in this protocol in comparison to pyridine and 1,10-phenanthroline as ligand. Thus the optimum yield with complete regioselectivity to obtain the desired product (3e), was attained by using 2 equiv. of Ag_2_CO_3_ as an additive, and 30 mol% of 2,6-lutidine in presence of the catalyst 5 mol% of Pd(OAc)_2_ for 16 h at 120 °C. In a mixed solvent of DMSO and dioxane. The scope of cross-coupling reaction between various other pyridine *N*-oxides containing fluoro, methyl, benzyloxyl and methoxy groups *etc.* with triazole ring bearing *o*-tolylbenzyl, *p*-methoxylbenzyl, *p*-fluorobenzyl, *m*-tolyl-benzyl, *etc.* at *N*-1 position was also analyzed and the corresponding cross-coupled products were obtained in good yields (65–84%), however, no homocoupling product was observed (Scheme 11).

**Scheme 12:**
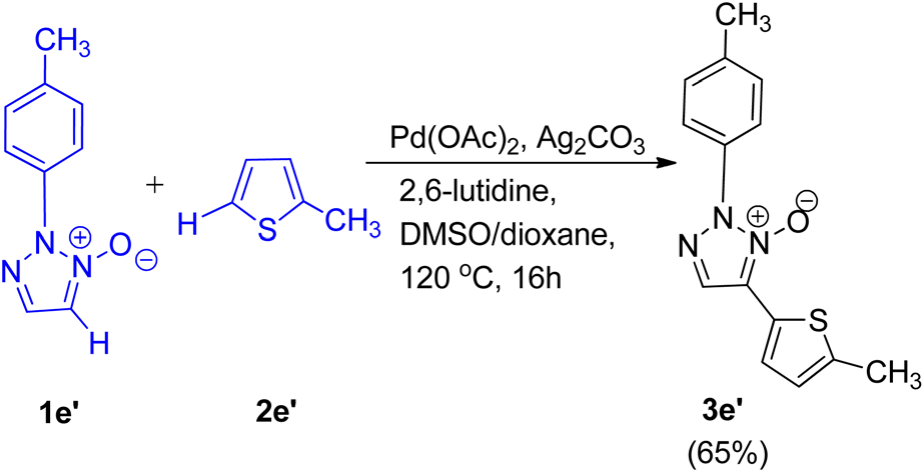
C–H/C–H oxidative cross-coupling between 1,2,3-triazole *N*-oxides and 2-methylthiophene.

C–H/C–H oxidative cross-coupling reaction of 1,2,3-triazole *N*-oxides (1e′) with 2-methylthiophene (2e′) (Scheme 12) was also found to undergo to completion to give the expected product (3e′) with 65% yield under similar conditions. Control experiments were performed to gain an insight into the reaction mechanism and it was proposed that this cross-coupling reaction follows a catalytic cycle, similar to the conventional mechanism being followed during the pyridine *N*-oxide cross-coupling reaction.^11^ However further studies are needed to completely gain an understanding of the mechanism.

**Scheme 13:**
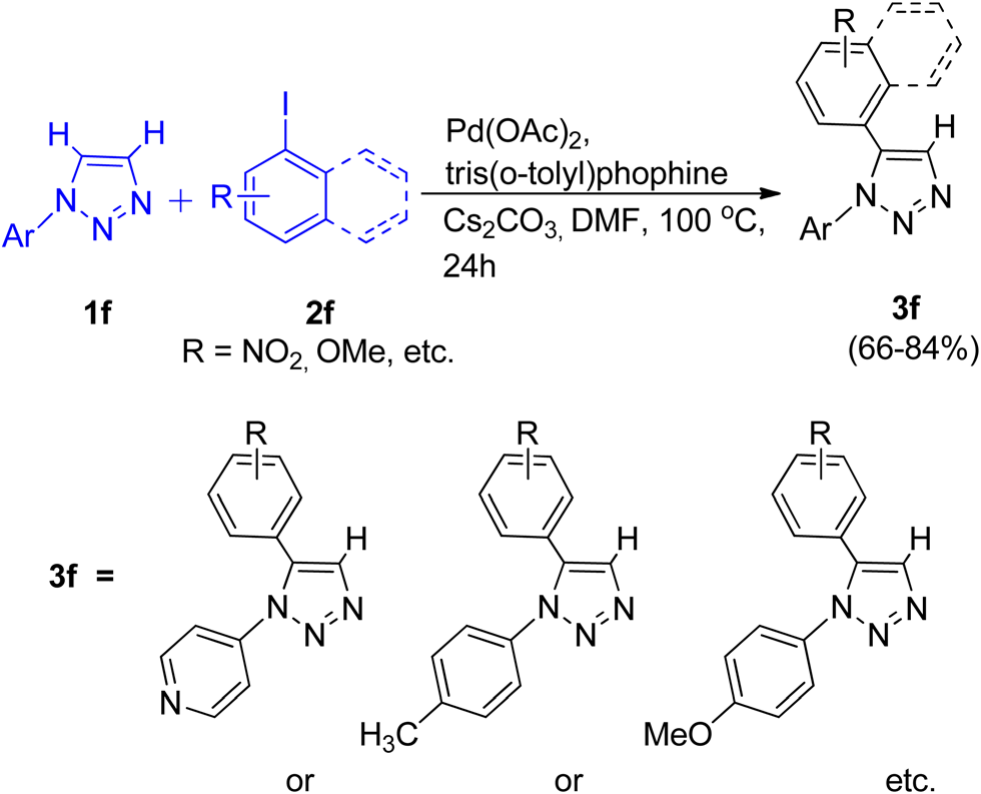
C-5 arylation of *N*-aryl substituted 1,2,3-triazole.

A direct palladium catalyzed arylation reaction between *N*-aryl 1,2,3-triazole (1f) and aryl-halide (2f) for the formation of C-5 substituted *N*-aryl 1,2,3-triazole was reported in 2015 by Mahendra Patil and co-workers.^12^ The usual PPh_3_ ligand was found to be less effective as compared to the bulkier tris(*o*-tolyl)phosphine ligand in this protocol. Upon using K_2_CO_3_ instead of Cs_2_CO_3_ the yield of the reaction was not affected however the reaction rate dropped. Thus in an inert atmosphere, the optimum conditions for the reaction involved utilizing Pd(OAc)_2_ (10 mol%) as a catalyst, Cs_2_CO_3_ (2.0 equiv.) as base, tris(*o*-tolyl)phosphine (20 mol%) as ligand, in DMF at 100 °C for 24 h (Scheme 13).

The electronic environment caused by the substituents present on *N*-aryl 1,2,3-triazoles did not affect the yield of desired product. By employing the above given conditions, C-5 substituted *N*-aryl 1,2,3-triazole were formed in moderate to good yield (66–84%) from arylation reaction of *N*-aryl 1,2,3-triazole. The catalytic cycle proposed for this protocol involves a concerted metalation deprotonation (CMD) pathway.

**Scheme 14:**
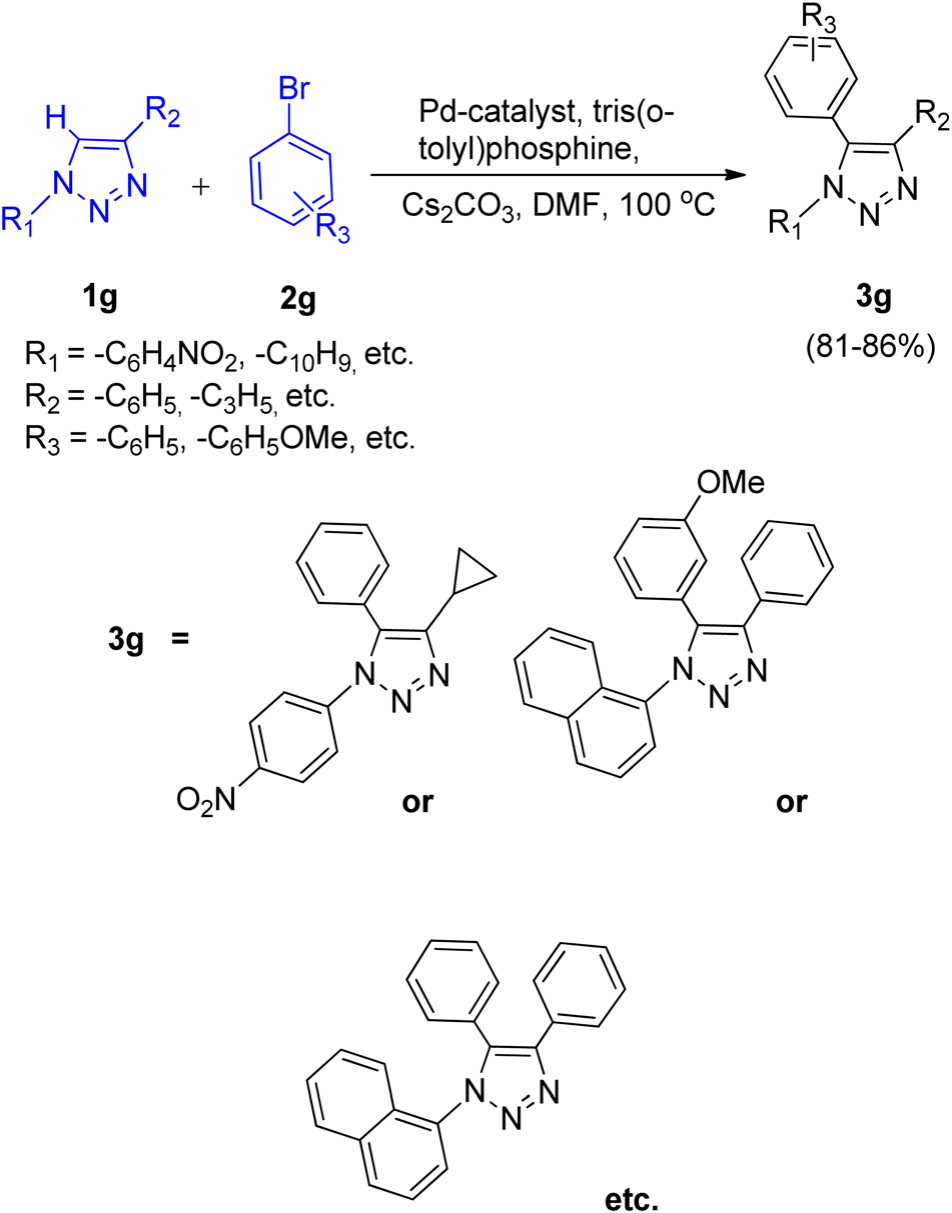
C-5 arylation of 1,4-disubstituted 1,2,3-triazole.

This protocol was also shown to be applicable for the arylation of 1,4-disubstituted 1,2,3-triazole (1g) on applying the same reaction conditions resulting in good yields (81–86%) of the desired product (Scheme 14).

**Scheme 15:**
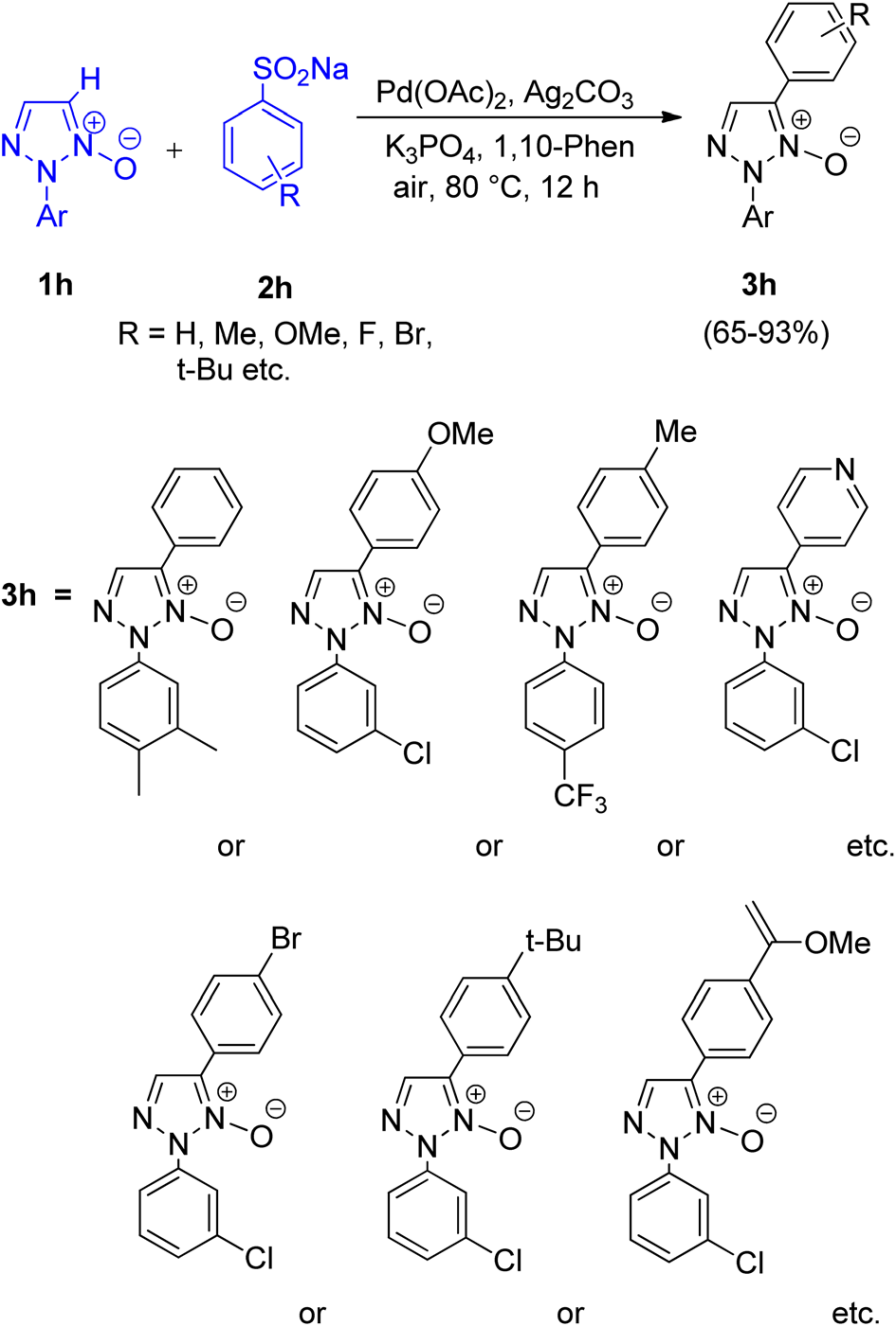
Arylation of 1,2,3-triazole *N*-oxides using sodium arenesulfinates.

A protocol allowing the use of arylsulfinic acids and salts as arylating agents (2h) in order to perform direct C-5 arylation of 1,2,3-triazole *N*-oxides (1h) *via* desulfitation was developed by Qing Huang, Liangxian Liu and co-workers in 2015.^13^ For this protocol various catalysts such as CuI, Cu(OAc)_2_, AgNO_3_, NiSO_4_, and Pd(OAc)_2_ were analysed, however except Pd(OAc)_2_, others showed no reactivity. Among the various oxidants (AgOAc, AgNO_3_, Ag_2_CO_3_ and AgBF_4_), solvents (*t*-BuOH, toluene, 1,4-dioxane, DMF and DME), ligands (1,10-phenanthroline and 4,4′-bipyridine), and bases (Na_2_CO_3_, Cs_2_CO_3_, KOt–Bu and K_3_PO_4_) analyzed, Ag_2_CO_3_ as an oxidant, a combination of DME–DMF as solvent 1,10-phenanthroline as ligand and K_3_PO_4_ as base worked efficiently. Thus the optimized reaction condition includes 5 mol% palladium, 1 equiv. of silver carbonate, 2 equiv. of tri-potassium phosphate and 40 mol% 1,10-phenanthroline at 80 °C for 12 h in a mixed solvent of DME–DMF (Scheme 15).

The presence of both electron rich and electron-deficient groups and a number of functional groups (fluoro, chloro, trifluoromethyl, and methyl *etc.*) on triazole ring were tolerated under optimum conditions giving good to excellent yield (65–93%). However, the presence of electron poor aryl groups at the *N*-2 position triazole ring gave better yield in comparison to the electron rich counterpart.

**Fig. 3:**
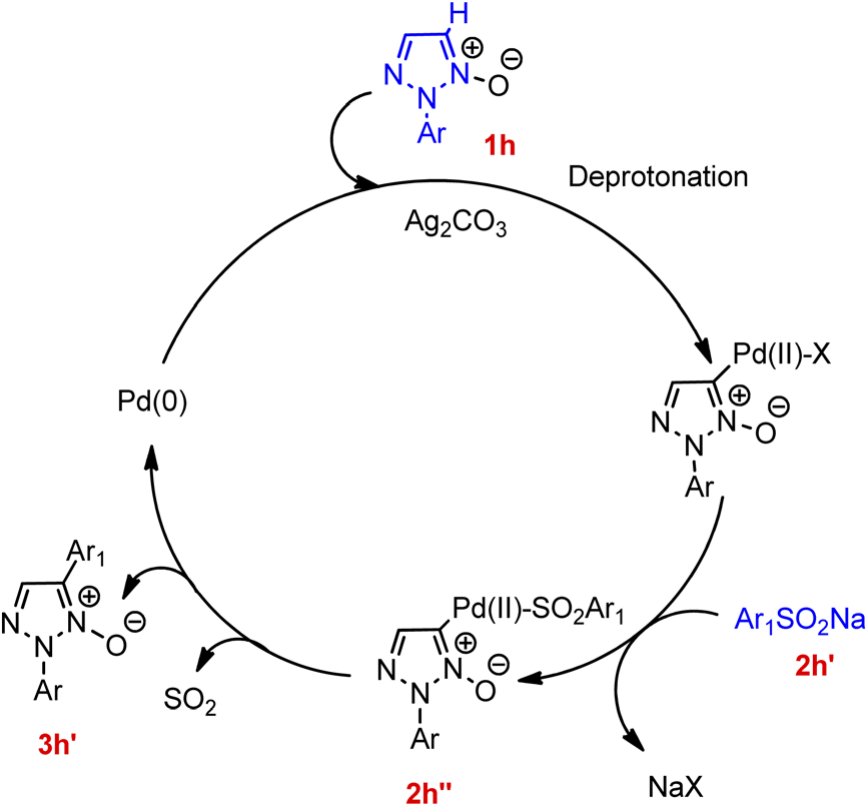
Proposed mechanism for the palladium-catalyzed arylation between of 1,2,3-triazole *N*-oxide and sodium arylsulfinate.

Contrary to this, in the case of reaction between 2-substituted 1,2,3-triazole *N*-oxides with various aryl sulfinic acid sodium salts (such as sodium 4-bromobenzenesulfinate, sodium 2-fluorobenzenesulfinate, sodium naphthalene-2-sulfinate, sodium 4-*tert*-butylbenzenesulfinate, sodium pyridine-4-sulfinate, and sodium 4-(methoxycarbonyl)benzenesulfinate, *etc.*) though the presence of many functional groups on aryl sulfinic acid sodium salts were tolerated, aryl sulfinic acid sodium salts bearing electron-donating groups resulted in a slightly better yield of the desired product than electron-withdrawing groups. Various controlled experiments were performed in order to get a better insight of the mechanism (Fig. 3), indicated that the cleavage of C-5-H of triazole ring (1h) initiates the arylation reaction, so that the palladium catalyst can react with deprotonated 1,2,3-triazole *N*-oxide to generate an intermediate. This palladium coordinated species reacts with incoming sodium arylsulfinate to form an intermediate 2h′′ which then ultimately undergoes desulfitation followed by reductive elimination to afford the final arylated product 3h′ with subsequent oxidation of the palladium(0) species for the regeneration of the palladium(ii) catalyst in order to complete the catalytic cycle.

This operationally simple, highly regioselective protocol with high halogen compatibility of the process, can also be applied to a wide range of coupling partners including sodium arylsulfinates, triphenylphosphine, and diphenylphosphine oxide.

**Scheme 16:**
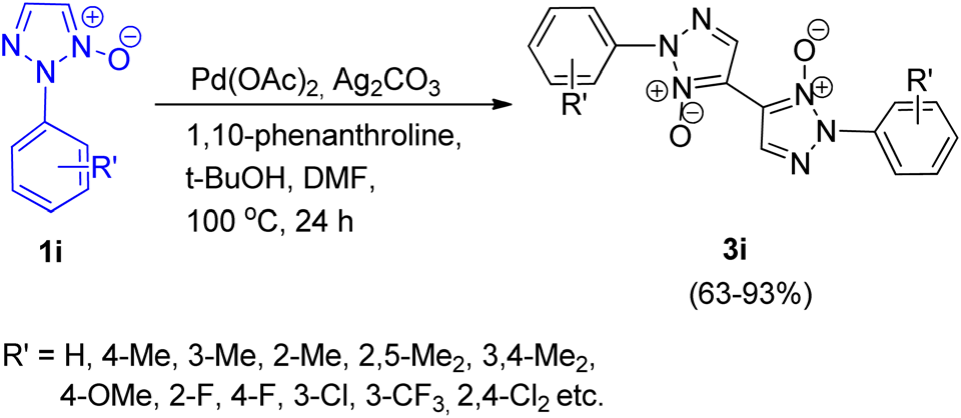
Oxidative homocoupling of 1,2,3-triazole *N*-oxides.

An efficient method for palladium catalyzed homocoupling of 1,2,3-triazole *N*-oxides (1i) to produce bistriazole *N*-oxide (5,5′-linkage) with excellent regioselectivity was developed by Jiayi Zhu, Liangxian Liu and co-workers in 2016.^14^ Various metal catalysts were analysed to attain the optimized condition such as Cu(OAc)_2_, FeCl_3_, AgNO_3_, NiSO_4_, and Pd(OAc)_2_, however none of them were able to produce the desired product in good yields except Pd(OAc)_2_. Similarly apart from DMF, usage of other solvents such as toluene, 1,4-dioxane, and *t*-BuOH, resulted in poor yield of the product. Although oxidants such as AgNO_2_, AgOAc, AgBF_4_, and AgNO_3_, gave 3i in 15–25% yield, utilizing Ag_2_CO_3_ increased the yield upto 85%. Among various bases screened such as Na_2_CO_3_, Cs_2_CO_3_, LiOH, and *t*-BuOK, *t*-BuOK proved to be the most effective base. Thus the optimized conditions for this protocol giving good result (85% yield) of 3i involved 5 mol% of Pd(OAc)_2_ as a catalyst, 1 equiv. of Ag_2_CO_3_ as an oxidant, 20 mol% 1,10-phenanthroline as a ligand with 1 equiv. of *t*-BuOK as base in DMF at 100 °C for 24 h (Scheme 16), however desired product was not obtained in the absence of palladium catalyst.

The electronic environment imposed by the aryl group present at *N*-2 position slightly affected the yield of the reaction. A varied number of substituted 1,2,3-triazole *N*-oxides were analyzed and the reaction was found to proceed smoothly to yield the desired product. The reactant in this protocol also showed good compatibility with the presence of many functional groups. However, electron-deficient triazole substrates furnished a better yield (86–93%) of the homocoupling product than electron rich counterparts (63–85%). Based on previous literature and some experiments performed in order to get an insight into the mechanism of this transformation, a plausible mechanism was given which suggested that the homocoupling was initiated by the cleavage of the C-5-H of 1i. The mechanism follows a similar pathway as given by Chunxiang Kuang *et al.* in 2013 for the palladium catalyzed homocoupling reaction of 2-substituted 1,2,3-triazole *N*-oxides.^9^

This was an operationally simple, highly regioselective method for the formation of bistriazole *N*-oxide allowing the isolation of the desired product in good to excellent yield.

**Scheme 17:**
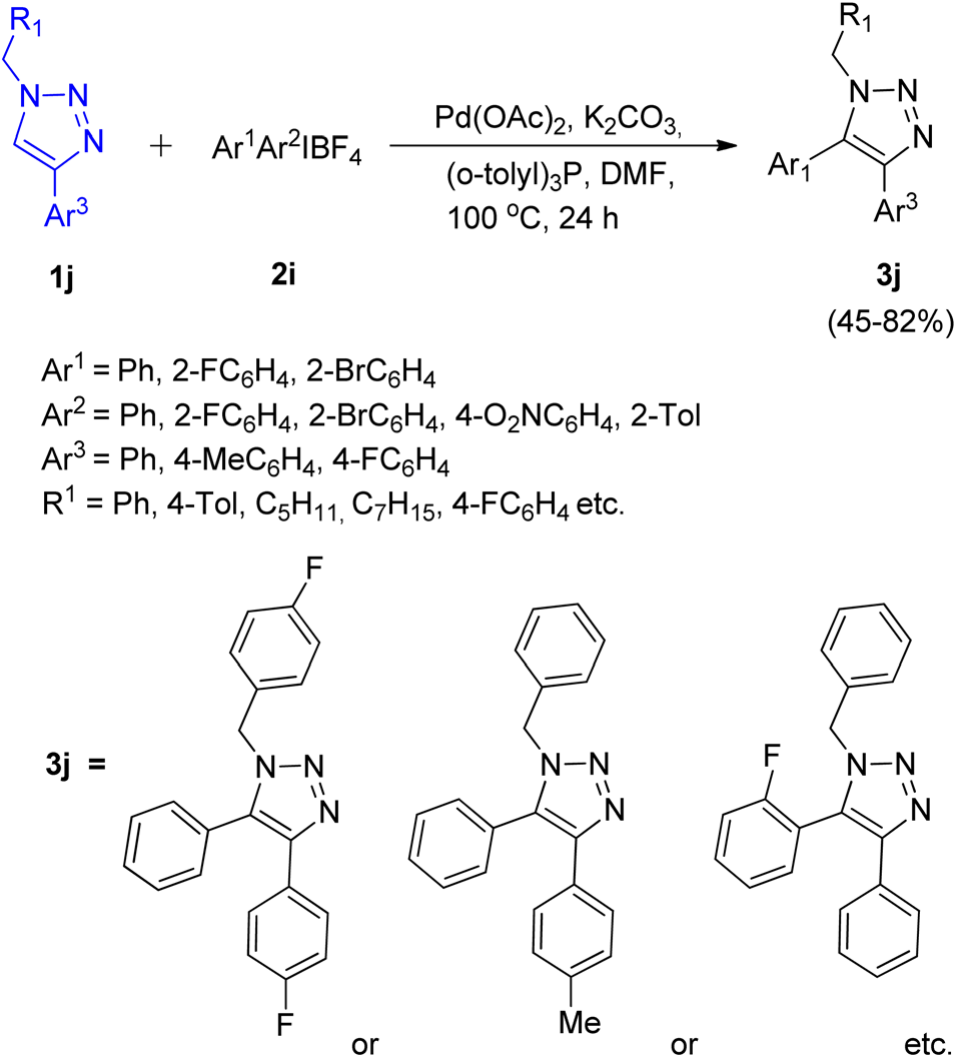
C-5 arylation of 1,4-substituted 1,2,3-triazoles using diaryliodonium salt.

**Scheme 18:**
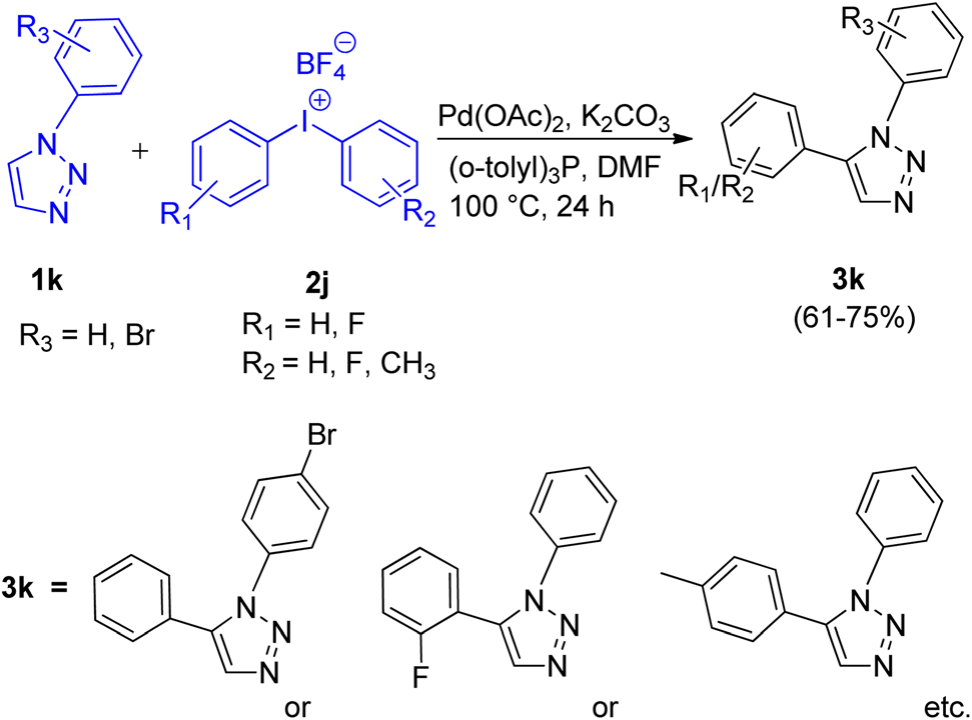
Regioselective C-5 arylation of 1-substituted triazoles using diaryliodonium salt.

First report for the C–H arylation of 1,2,3-triazoles using diaryliodonium salt (2i) as arylating agent came in 2017 by Zhengyin Du and Hua Feng *et al.*^15^ C-5 arylation of 1,4-disubstituted 1,2,3-triazoles (Scheme 17; 1j) as well as of 1-substituted 1,2,3-triazoles (Scheme 18; 1k) were analysed under several conditions to obtain the optimized condition. Among transition metal catalysts which were analysed such as CuI, CuNPs, PdI_2_, Pd(OAc)_2_ and Pd(PPh_3_)_2_Cl_2_, as compared to Pd(OAc)_2,_ all of them proved to be inefficient for this protocol. Similarly among various phosphine ligand analysed such as (*o*-tolyl)_3_P, PPh_3_, tricyclohexylphosphine (PCy_3_), 2-(dicyclohexylphosphino)-2′,4′,6′-triisopropylbiphenyl (X-Phos), and (*o*-tolyl)_3_P worked best for this transformation. Thus the optimized conditions gave promising results by employing 5 mol% Pd(OAc)_2_ as catalyst, 2 equiv. K_2_CO_3_ as base, 10 mol% (*o*-tolyl)_3_P as a ligand, in DMF at 100 °C for 24 h. The given protocol was able to generate the desired product in as high as 82% of the isolated yield.

The C-5 arylation of 1-substituted 1,2,3-triazole (1k) proceeded with higher regioselectivity, however, both substituted triazoles required the presence of palladium catalyst in DMF to provide the desired product. The presence of oxygen was proven insignificant by conducting the experiment under argon atmosphere which gave 80% yield of the required product. The electronic environment of substituents present on benzene ring of triazole played a small role in the reaction. However when R_1_ attached to 1j was an alkyl group instead of an aryl group, moderate yield of the arylated product was obtained (53–66%). Similarly, on analysing various diaryliodonium salts (2i) for C-5 arylation of 1,4-substituted 1,2,3-triazoles, up to 53–82% yield of the corresponding arylated product was obtained on employing symmetrical di(2-fluorophenyl)iodonium tetrafluoroborate and di(2-bromophenyl)iodonium tetrafluoroborate, however, on utilizing unsymmetrical diaryliodonium salts, competitive arylation owing to the steric and electronic effect imposed by substituents present on aryl group of diaryliodonium salt was observed with 45–71% yield. Opposing observations were made for C-5 arylation of 1-substituted triazoles using symmetrical and unsymmetrical diaryliodonium salts (2j), where symmetrical diaryliodonium salts gave arylated product in moderate yield of 61–67% whereas unsymmetrical diaryliodonium salts gave the arylated product in total yield of 72–75%.

This protocol enabled the synthesis of several trisubstituted triazoles in high yields due to its good functional group tolerance.

**Scheme 19:**
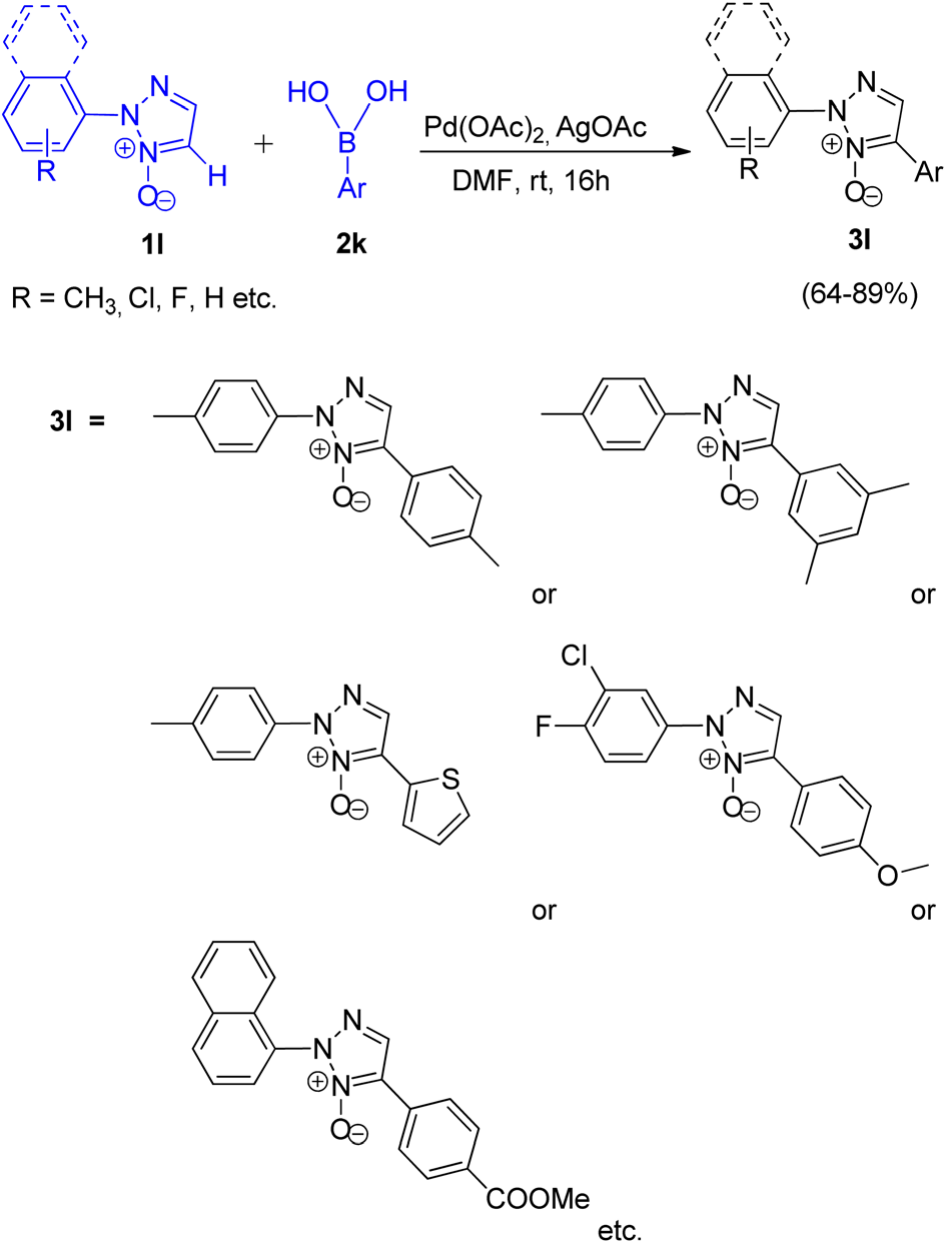
C-5 arylation of 2-aryl-1,2,3-triazole *N*-oxides by utilising arylboronic acids.

Wei Liu and co-workers (2017)^16^ reported a simple method for synthesizing 2,4-disubstituted 1,2,3-triazole (3l) moiety by utilising the palladium catalyzed direct regioselective arylation reaction between Ar-B(OH)_2_ (2k) and 2-aryl-1,2,3-triazole *N*-oxides (Scheme 19; 1l).

Screening experiments were performed to get an insight into the optimized reaction conditions revealed that AgOAc acts more efficiently as an oxidant in comparison to others such as CuSO_4_, Ag_2_O, Ag_2_CO_3_, AgNO_3_, and Ag_2_SO_4_. Similarly, the reaction proceeded with higher yield of the desired product when it was carried out in DMF rather than in DMSO, 1,4-dioxane and toluene. One of the major advantage of this protocol is its ability to undergo desired conversion at room temperature giving about 89% of the arylated product. On increasing the temperature to 70 °C, not only did the yield of arylated product gets reduced to 23%, but a biphenyl product with upto 45% yield also gets formed. On increasing the temperature to 100 °C, biphenyl was obtained in very high yield of 95%. Thus the optimized conditions for the given protocol employs 5.0 mol% of Pd(OAc)_2_ as catalyst, 5.0 mol% AgOAc as oxidant in DMF as solvent at rt, for 16 h, under air resulting in upto 89% of the desired arylated product.

**Scheme 20:**
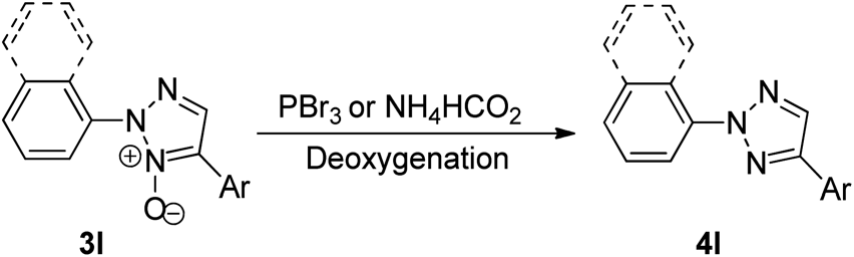
Deoxygenation of 2,4-disubstituted-1,2,3-triazole *N*-oxides (3l).

Thus this protocol requires mild reaction conditions at an ambient temperature, which has also widened the applicability of this protocol. On analysing the scope of this reaction, aryl boronic acids containing both electron-rich and electron poor groups gave moderate to good yields (64–89%) of the desired product including thiopheneboronic acids. A plausible mechanism for this protocol has been proposed to occur similar to the usual direct arylation reaction of pyridine *N*-oxides using arylboronic acids.^16^ The deoxygenation of the arylated derivative of 2-aryl-1,2,3-triazole *N*-oxides (3l) through palladium catalyzed reduction using PBr_3_ and ammonium formate gives the desired product (Scheme 20; 4l).

**Scheme 21:**
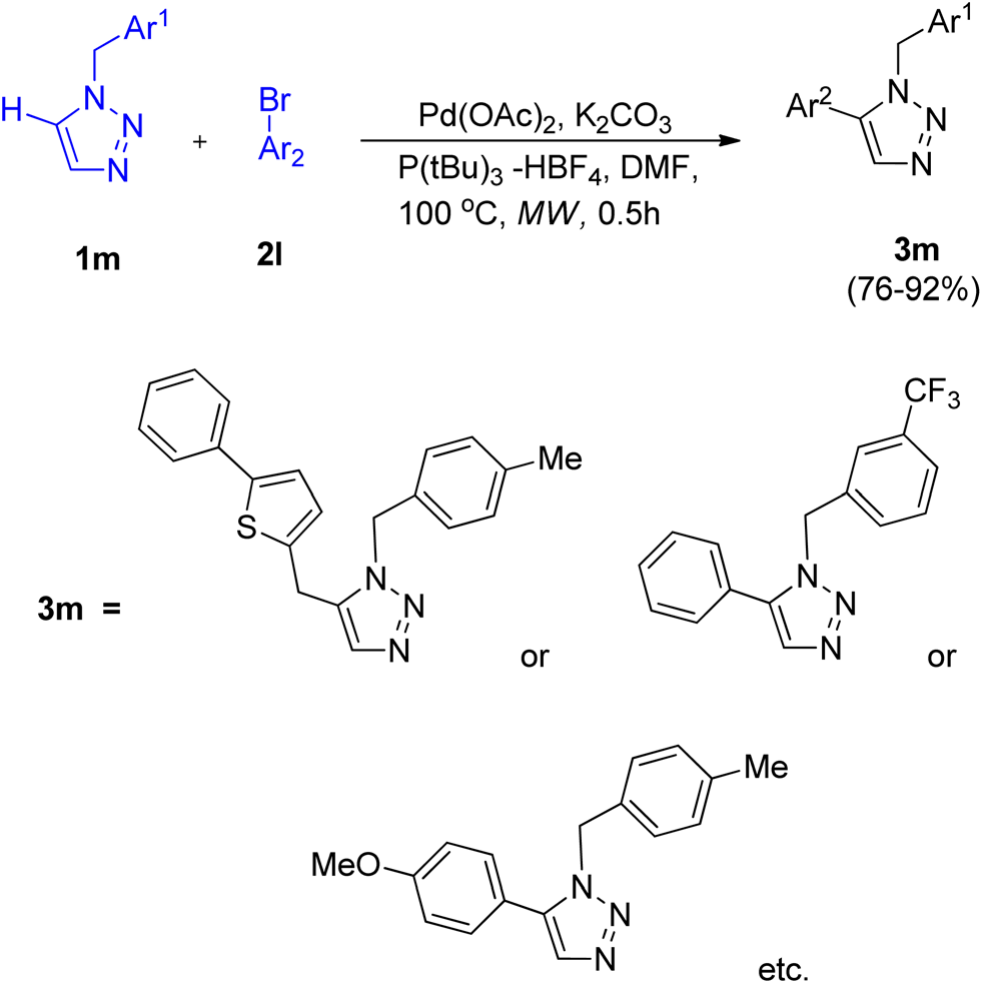
Microwave assisted direct C-5 arylation of 1-benzyl-1,2,3-triazole.

After the development of cross-coupling reaction by Chunxiang Kuang and co-workers (2014) involving 1-benzyl-1,2,3-triazoles, Wei Liu *et al.* in 2019 (ref. 17) gave microwave assisted efficient pathway for the direct arylation of 1-benzyl-1,2,3-triazole (1m) using Ar-Br (2l) in order to generate 1,5-disubstituted 1,2,3-triazoles (3m) in 76–92% yield (Scheme 21).

Numerous screening experiments were performed in order to obtain the optimized condition for this reaction. These experiments showed that among DBU, pyridine, Et_3_N and K_2_CO_3_, K_2_CO_3_ as base worked best and among solvents such as DMF, 1,4-dioxane, DMSO and toluene, DMF was the most efficient solvent. Thus under an atmosphere of N_2_ in the presence of Pd(OAc)_2_ (5.0 mol%) as catalyst, 5.0 mol% P(tBu)_3_ -HBF_4_ as ligand, K_2_CO_3_ (1.0 mmol) as base, and DMF as solvent, at 100 °C for 0.5 h in a microwave the reaction gave highest yield of the products.

Apart from aryl bromide, aryl chlorides were also investigated as an arylating agent, however arylation failed to occur. Except for 2-bromopyridine and 2-bromothiophene, which could not produce the intended product, the given protocol is applicable to a broad range of substituted triazoles and aryl halides containing both electron withdrawing and electron donating groups. The proposed mechanism involved a concerted metalation-deprotonation (CMD) mechanism for the C–H activation step, however further studies are needed to completely understand the mechanism. This protocol provides a better alternative for the preparation of 1-benzyl-5-aryl-1,2,3-triazoles in good yield as compared to the previous ones.

## 3. Copper catalyzed C–H functionalization on triazole rings

### 3.1. Copper catalyzed C–H functionalization on 1,2,3-triazole rings

**Scheme 22:**
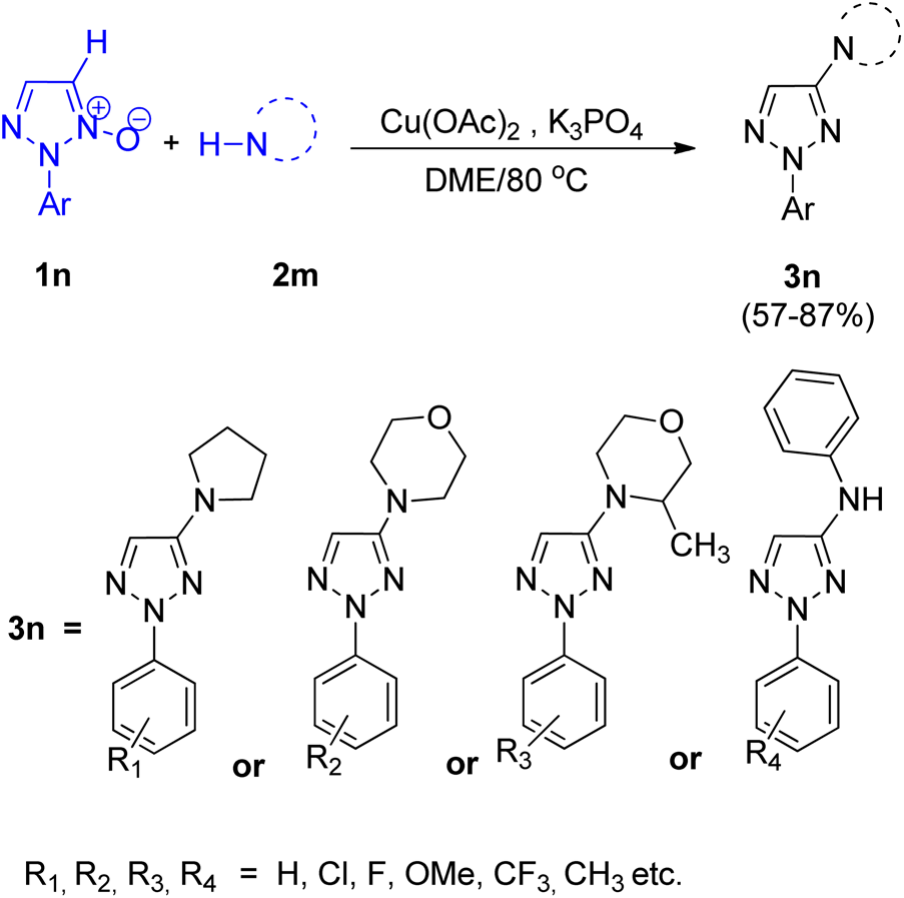
Copper-catalyzed amination of 1,2,3-triazole *N*-oxides.

Zhengwang Chen, and Liangxian Liu^18^ in 2015 reported an efficient approach for copper catalyzed direct synthesis of 4-amino-2-aryl-1,2,3-triazole derivatives (3n). The protocol required mild conditions for direct C–H amination of 2-aryl-1,2,3-triazole *N*-oxides for the development of 4-amino-2-aryl-1,2,3-triazole derivatives. Effective coupling partners included various primary as well as secondary amines. In order to attain the optimized reaction condition, various screening experiments were performed and it was found that the choice of solvent plays a major role in product yield. In this regard, DME was found to result in the highest yield of desired product, on the other hand, the reaction was completely suppressed upon usage of *N*,*N*-dimethylformamide (DMF). Among several catalysts (such as CuCl_2_, CuSO_4_, CuI and other palladium and silver salts) analysed, Cu(OAc)_2_ showed the best performance with highest efficiency. The optimized CDC (cross-deprotonative coupling) reaction between 2-aryl-1,2,3-triazole *N*-oxide (1n) and an amine source (2m) included inexpensive and environment friendly Cu(OAc)_2_ (20 mol%) as catalyst, with 2 equiv. of K_3_PO_4_ in DME at 80 °C for 12 h (Scheme 22).

**Fig. 4:**
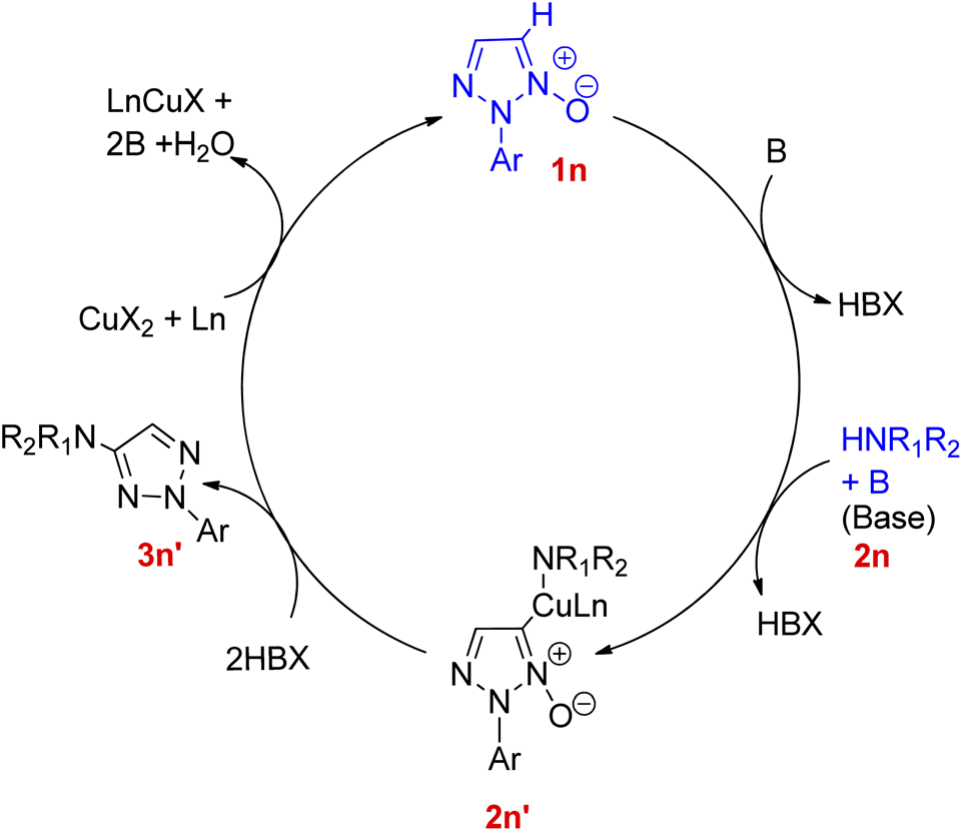
Plausible mechanism for copper-catalyzed amination of 2-aryl-1,2,3-triazole *N*-oxides (1n).

On analysing the scope of the substrate, perfect regioselectivity with up to 82% of the yield was observed during the amination of halogen or alkyl-substituted 2-aryl-1,2,3-triazole *N*-oxides (1n). A plausible mechanism for copper-catalyzed direct coupling of 2-aryl-1,2,3-triazole *N*-oxide with amine given by them is shown in Fig. 4. This copper catalyzed C–N coupling begins with copper coordination at C-5 of 2-aryl-1,2,3-triazole *N*-oxides (1n) by removal of C-5 hydrogen. This organocopper species thus formed reacts with amine (2n) to generate the intermediate 2n′ which on subsequent reductive elimination produces aminated product 3n′ and generates a copper species in its lower oxidation state which then gets oxidized to a Cu(ii) moiety in order to complete the catalytic cycle. Since the cleavage of N^+^–O^−^ bond is observed during the catalytic cycle, thus further deoxygenation step is not required.

2-Aryl-1,2,3-triazole *N*-oxides bearing electron-withdrawing groups were found to be more reactive than the ones with electron-donating groups however, in both two cases, 2-substituted 1,2,3-triazole *N*-oxides gave the desired product in moderate to excellent yield (57–87%). Thus, substituted groups, and steric hindrance created by them, do not pose any significant effect on the reaction.

### 3.2. Copper catalyzed C–H functionalization on 1,2,4-triazole rings

**Scheme 23:**
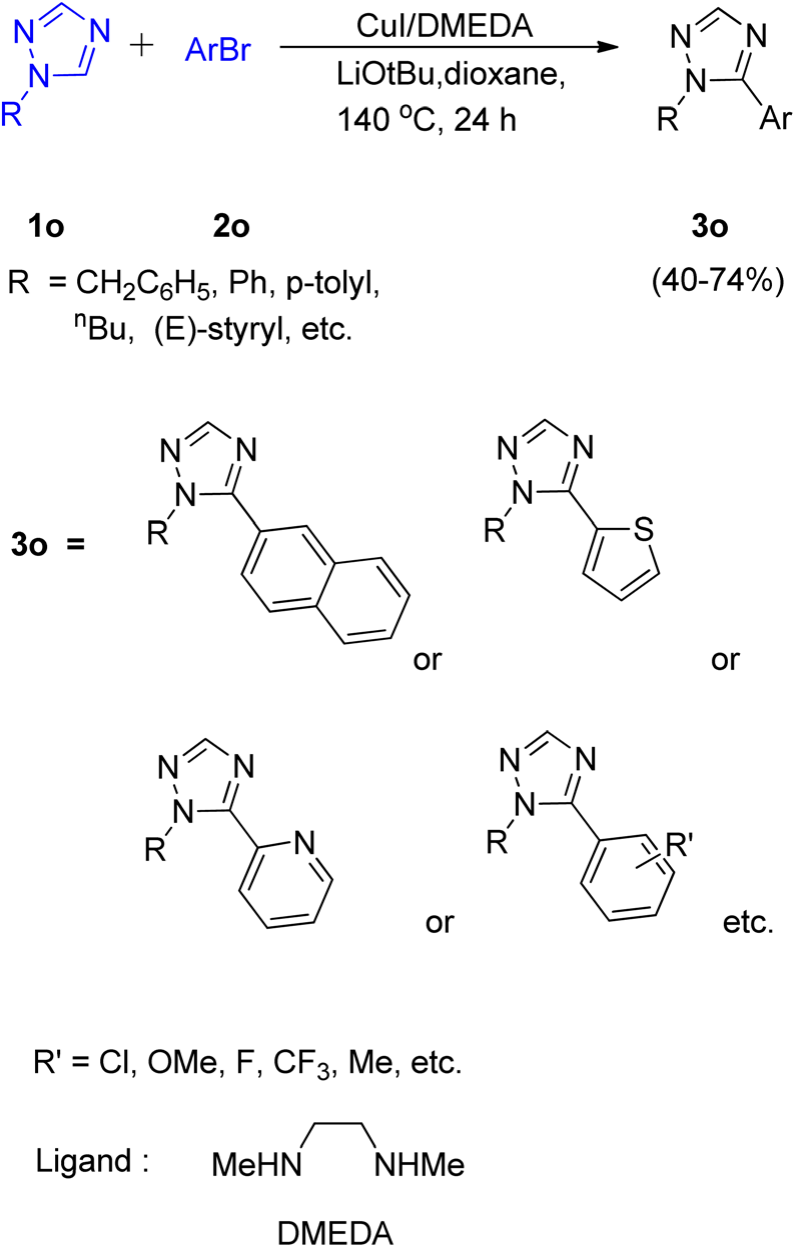
Copper catalyzed C–H arylation of 1,2,4-triazole ring using aryl bromides.

A convenient method utilizing Cu-diamine catalytic system for the C–H bond functionalization of easily accessible and inexpensive substrates *i.e.* simple 1,2,4-triazole ring (Scheme 23; 1o) was developed by Yong-Chua Teo *et al.* in 2016.^19^ In order to obtain the optimized conditions, various reaction parameters were screened. Solvent and bases such as DMF, ^*t*^AmylOH and K_3_PO_4_, K_2_CO_3_ proved to be ineffective as compared to dioxane (solvent) and LiO*t*Bu (base) since only trace amount of product was obtained in each of these attempts. The exploitation of Cu(I) sources other than CuI, such as CuBr and CuCl as catalysts proved to be insignificant with respect to improving the yield of this reaction. Thus with the optimal conditions involving 20 mol% CuI/DMEDA catalytic system with dioxane as solvent and 2.0 equiv. LiOtBu as base at 140 °C for 24 h, a good yield (up to 74%) of the desired product (3o) was obtained.

Most of the substituted 1,2,4-triazoles tested with the optimized reaction conditions gave the intended product. The occurrence of deprotonation at C-5 in the triazole ring is attributed to its higher reactivity at C-5 due to the more electron deficient character than other rings.

**Fig. 5:**
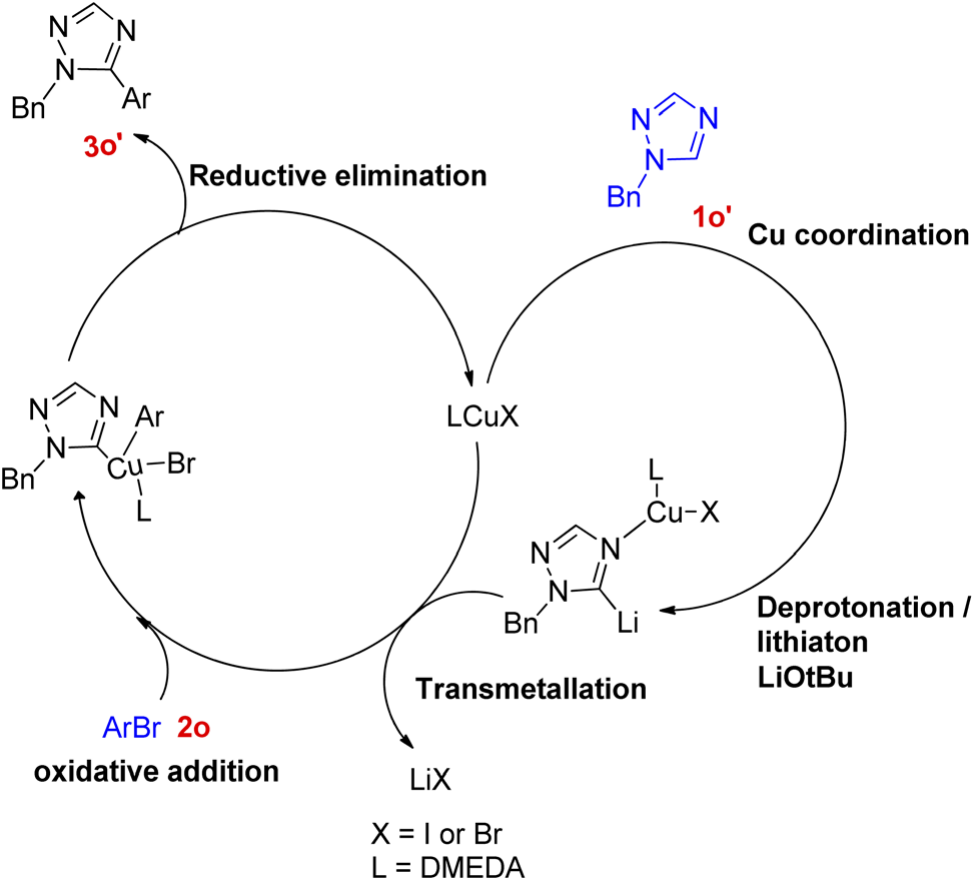
Plausible reaction mechanism for arylation of 1,2,4-triazole.

On analysing various aryl bromides for the arylation with 1o, their electronic effect and steric factor were found to play a major role in reaction efficiency as slightly lower arylation efficiency was observed with electron poor aryl bromides, however the reaction showed good compatibility with presence of chloro and fluoro groups on arylating agent. A plausible mechanism is shown in Fig. 5, which starts with copper coordination to *N*-4 position of the ring 1o′ which then assist in the *tert*-butoxide deprotonation at C-5 followed by C-5 lithiation step. The resulting complex undergoes transmetallation followed by oxidative addition/coupling with an aryl bromide. The coupled complex thus formed undergoes a reductive elimination step to form the desired arylated product 3o′ and regenerate the catalyst.

## 4. Conclusions

Due to their unique properties and applications in materials science^2^ and medicinal chemistry,^1^ triazoles have been investigated thoroughly in the last two decades. Various substituted triazole derivatives have been successfully synthesised using direct dehydrogenative coupling by the cleavage of C–H bonds. Taking note of the increasing significance of triazoles along with the sustainable nature of C–H functionalization, we believe that these methods for synthesis of more substituted triazoles through C–H activation will provide a more atom-economical, environmentally friendly way and an extra encouragement for the exploration of this growing area of research. Despite the development of these elegant reactions, there is still a large scope and an intrinsic requirement to develop bistriazole compounds which should be explored in future.

## Conflicts of interest

There are no conflicts to declare.

## Supplementary Material
